# Imaging modalities for characterising T1 renal tumours: A systematic review and meta‐analysis of diagnostic accuracy

**DOI:** 10.1002/bco2.355

**Published:** 2024-06-21

**Authors:** Hannah Warren, Jack B. Fanshawe, Valerie Mok, Priyanka Iyer, Vinson Wai‐Shun Chan, Richard Hesketh, Eleanor Zimmermann, Veeru Kasivisvanathan, Mark Emberton, Maxine G. B. Tran, Kurinchi Gurusamy

**Affiliations:** ^1^ Division of Surgery and Interventional Science University College London London UK; ^2^ Royal Free Hospital Specialist Centre for Kidney Cancer London UK; ^3^ Guy's & St Thomas' NHS Foundation Trust London UK; ^4^ Faculty of Medicine University of British Columbia Vancouver Canada; ^5^ Guy's, King's and St Thomas' School of Medical Education King's College London London UK; ^6^ School of Medicine University of Leeds Leeds UK; ^7^ Centre of Medical Imaging A University College London London UK; ^8^ University Hospitals Plymouth Plymouth UK

**Keywords:** diagnostic accuracy, imaging, renal tumours

## Abstract

**Objectives:**

International guidelines recommend resection of suspected localised renal cell carcinoma (RCC), with surgical series showing benign pathology in 30%. Non‐invasive diagnostic tests to differentiate benign from malignant tumours are an unmet need. Our objective was to determine diagnostic accuracy of imaging modalities for detecting cancer in T1 renal tumours.

**Methods:**

A systematic review was performed for reports of diagnostic accuracy of any imaging test compared to a reference standard of histopathology for T1 renal masses, from inception until January 2023. Twenty‐seven publications (including 2277 tumours in 2044 participants) were included in the systematic review, and nine in the meta‐analysis.

**Results:**

Forest plots of sensitivity and specificity were produced for CT (seven records, 1118 participants), contrast‐enhanced ultrasound (seven records, 197 participants), [^99m^Tc]Tc‐sestamibi SPECT/CT (five records, 263 participants), MRI (three records, 220 participants), [^18^F]FDG PET (four records, 43 participants), [^68^Ga]Ga‐PSMA‐11 PET (one record, 27 participants) and [^111^In]In‐girentuximab SPECT/CT (one record, eight participants). Meta‐analysis returned summary estimates of sensitivity and specificity for [^99m^Tc]Tc‐sestamibi SPECT/CT of 88.6% (95% CI 82.7%–92.6%) and 77.0% (95% CI 63.0%–86.9%) and for [^18^F]FDG PET 53.5% (95% CI 1.6%–98.8%) and 62.5% (95% CI 14.0%–94.5%), respectively. A comparison hierarchical summary receiver operating characteristic (HSROC) model did not converge. Meta‐analysis was not performed for other imaging due to different thresholds for test positivity.

**Conclusion:**

The optimal imaging strategy for T1 renal masses is not clear. [^99m^Tc]Tc‐sestamibi SPECT/CT is an emerging tool, but further studies are required to inform its role in clinical practice. The field would benefit from standardisation of diagnostic thresholds for CT, MRI and contrast‐enhanced ultrasound to facilitate future meta‐analyses.

## 1 INTRODUCTION

Increasing use of cross‐sectional imaging has resulted in a rise in detection of incidental renal tumours. Current standard of care for T1 renal tumours, as defined by the Union for International Cancer Control,^1^ is surgical resection.^2^ However not all renal tumours are cancer, with up to 30% of partial nephrectomy specimens being benign.^3^ Partial or radical nephrectomy represents overtreatment of benign renal tumours and can be avoided if the distinction is made accurately before surgery.

Despite high diagnostic accuracy of renal tumour biopsy, it has not been widely adopted due to concerns about bleeding, tumour seeding, non‐diagnostic samples, difficulties in accessing anatomically complex tumours and assessment of only localised areas within the tumour.^4^ Diagnostic imaging therefore overcomes several important limitations of biopsy.

A recent descriptive review of novel imaging techniques for renal tumours concluded that [^99m^Tc]Tc‐sestamibi SPECT/CT and radiolabelled girentuximab are the closest to clinical adoption.^5^ However, the lack of quantitative analysis of diagnostic accuracy and how they compare to existing imaging techniques limits conclusions that can be drawn from the review.

In order to address the evidence gap, this systematic review was performed to determine and compare the diagnostic accuracy of various imaging modalities for detecting cancer in renal tumours.

## 2 METHODS

### 2.1 Protocol and registration

The protocol was developed according to PRISMA‐DTA^6^ and principles outlined in the Cochrane Handbook for systematic reviews of diagnostic accuracy v2,^7^ and prospectively registered with PROSPERO (CRD42022303473). Protocol deviations are summarised and justified in the protocol.

### 2.2 Eligibility criteria

Primary research articles evaluating the diagnostic accuracy of any imaging modality to characterise T1 renal tumours as malignant or benign as defined by a histopathological reference standard from surgery or biopsy were included. Prospective and retrospective studies were included. Studies that did not report sufficient diagnostic accuracy data, that is, the number of true and false positives and true and false negatives, were excluded. Studies that included participants with renal tumours of any stage were included if measures for T1 tumours could be extracted separately. Case–control studies were excluded as they are at high risk of bias. Full manuscripts and conference abstracts with sufficient information to meet the inclusion criteria were included.

### 2.3 Information sources

Comprehensive searches of electronic databases MEDLINE, EMBASE, Science Citation Index, The Cochrane Library, Clinicaltrials.gov and WHO trials register were performed from inception to 12 January 2023.

### 2.4 Search

Individual search strategies are detailed in Appendix S1. Due to the high number of texts during scoping searches (>40 000) we used a sensitivity‐maximising diagnostic filter to limit the results to a feasible number to review.^8^, ^9^ No language restrictions were applied.

Returned articles from each database were combined and duplicates removed using systematic review management software Covidence (available at covidence.org).

### 2.5 Study selection

Titles and abstracts were screened independently by two authors followed by full‐text screening in the same manner (JBF, VM, PI, VWSC, EZ or HW). Disagreements were discussed with a third author to reach consensus. Multiple publications from the same authors and institution with an overlapping recruitment period were managed by excluding the report with the smaller sample size. Reasons for exclusions were recorded. Hand searches of reference lists of included studies were performed to identify additional relevant literature. Non‐English language texts were translated to allow for screening and data extraction.

### 2.6 Data collection process

Data extraction was carried out independently by two authors from the research team (JBF, VM or PI) using a pre‐prepared and piloted form. Disagreements were reviewed and resolved by a third author (HW). Further information was sought from study authors where necessary.

### 2.7 Definitions for data extraction

The following data were extracted: study characteristics (authors, year of publication, institution, single or multi‐centre, country, language of publication, study period, study design, number of patients enrolled), patient characteristics (age, gender, ethnicity, number of tumours, lead tumour size, lead tumour volume), index test(s) (modality, manufacturer, model, specific settings, number of interpreters, presence of consensus interpretation, interpreter experience), reference standard(s) (modality, diagnostic criteria, number of interpreters, presence of consensus interpretation, interpreter experience), number of true positives, false positives, true negatives and false negatives. If data from multiple interpreters was presented, the results were averaged or the results from the authors' primary analysis were used. If results were reported at multiple thresholds, the diagnostic accuracy measures at each threshold were collected and the threshold used for the authors' primary analysis was used in our analysis. If studies explicitly stated that they had classified a malignant subtype of renal cell carcinoma (RCC) as benign due to indolent nature, we treated them as malignant in this review.

### 2.8 Risk of bias and applicability

Risk of bias and applicability concerns were assessed by two independent review authors (JBF, VM, PI) using the QUADAS‐2 tool and QUADAS‐C tools.^10^, ^11^ QUADAS‐2 and QUADAS‐C tools were customised to be relevant for this review (Appendix S2). Differences were resolved by a third author (HW).

### 2.9 Diagnostic accuracy measures

Sensitivity and specificity were reported as the principal measures of diagnostic accuracy. The unit of assessment was per lesion.

### 2.10 Synthesis of results and meta‐analysis

Study estimates of sensitivity and specificity were plotted on forest plots and receiver operating characteristic (ROC) space to explore between‐study variation in performance of each test. For imaging modalities with measures of diagnostic accuracy reported at the same threshold, bivariate analysis was attempted but convergence was not obtained. Therefore, univariate fixed‐effect model (determined by the model fit) was performed to calculate summary point estimates of sensitivity and specificity at that threshold.^12^ Comparison of these tests was attempted using a hierarchical summary receiver operating characteristic (HSROC) model, but convergence was not obtained. For imaging modalities reported at different positive thresholds, meta‐analysis was not performed as the result is clinically uninterpretable.^7^ When meta‐analysis was not performed, we reported the sensitivity and specificity with 95% confidence intervals from the individual studies, calculated with Review Manager version 5.4.1 (The Cochrane Collaboration, Software Update, Oxford, UK). Statistical analyses were performed with SAS v.9.4. The data and the code used for meta‐analysis are available from Appendix S3.

## 3 RESULTS

### 3.1 Study selection

**FIGURE 1:**
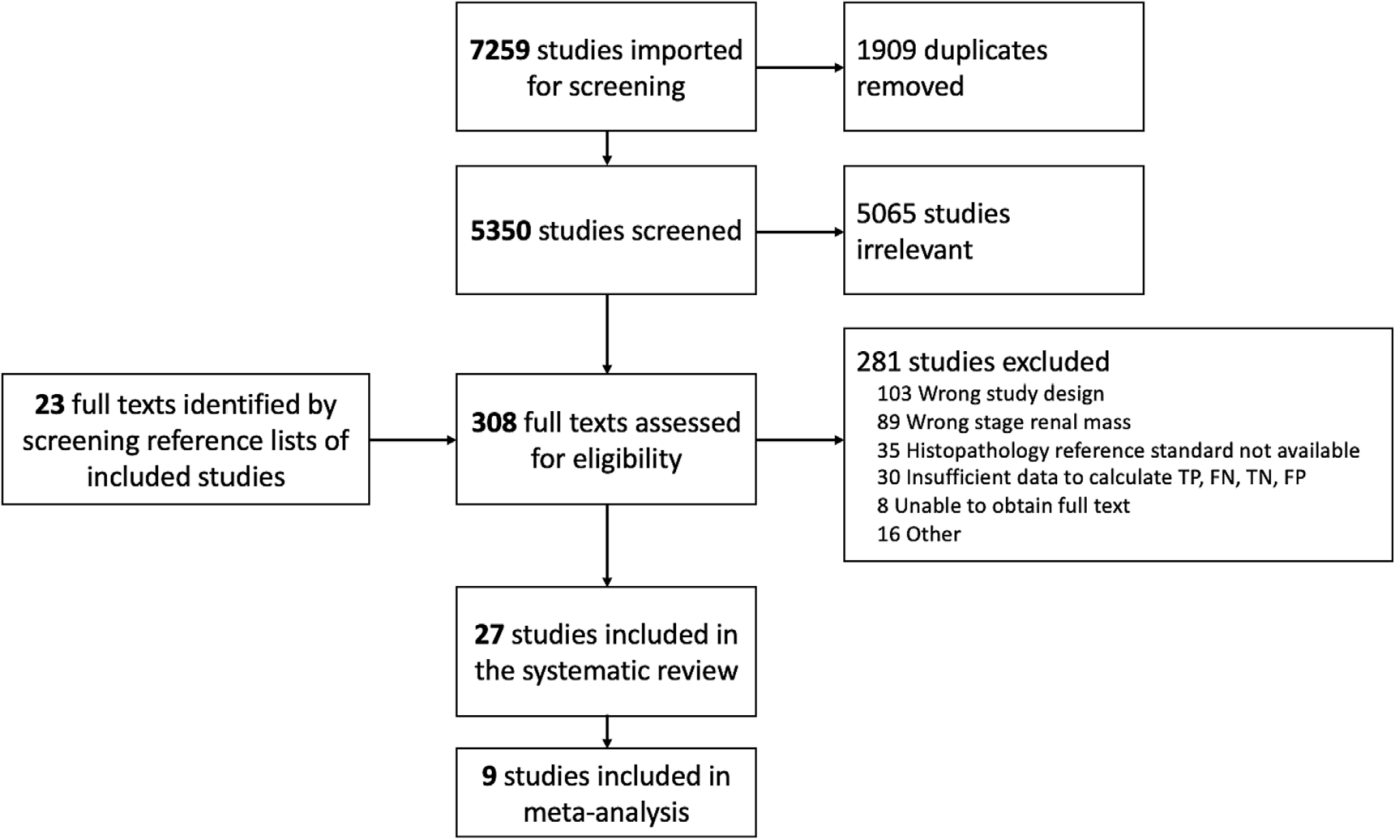
PRISMA flow diagram showing study selection process and reasons for exclusion from the meta‐analysis.

The search identified 5350 unique records following removal of duplicates. Of these, 5065 were excluded on title and abstract screening. An additional 23 references were identified through scanning reference lists of the identified studies, related search function and citing reference search. Of the resulting 308 references, 281 were excluded following full‐text review, with reasons stated in Figure 1. Twenty‐seven studies including 2277 tumours in 2044 patients were included. Nine studies with 314 lesions in 306 participants were included in the meta‐analysis of diagnostic accuracy of [^99m^Tc]Tc‐sestamibi SPECT/CT and [^18^F]FDG positron emission tomography (PET).

### 3.2 Study characteristics

Included studies reported the diagnostic accuracy of contrast‐enhanced computed tomography (CECT, seven studies), contrast‐enhanced ultrasound (CEUS, seven studies), [^99m^Tc]Tc‐sestamibi SPECT/CT (five studies), magnetic resonance imaging (MRI, three studies), [^18^F]FDG PET (four studies), [^68^Ga]Ga‐PSMA‐11 PET (one study) and [^111^In]In‐girentuximab SPECT/CT (one study). Individual study characteristics are reported in Table 1.

**TABLE 1:** Individual characteristics of included studies.

Study information			Patient selection								Index test	Reference standard	Data for analysis			
Study ID	Setting/country	Prospective or retrospective	Presentation	Sample size (participants)	Sample size (masses)	Females (%)	Average	Average tumour diameter (cm)	Tumour stage	Index test	Criteria for positive diagnosis	Histopathology reference standard	True positives	False positives	False negatives	True negatives
Grajo 2021	University hospital, USA	Retrospective	Patients undergoing surgery for <7 cm tumours	172	172	77 (45)	62	3.7	T1	CECT	Aorta‐lesion attenuation difference >24 HU	Surgical	79	13	27	14
Li 2004	Secondary or tertiary hospital, France	Retrospective	Patients undergoing evaluation for a solid renal tumour	161	161	48 (30)	66	NR	T1	CECT	Hypervascular, heterogenous, enhancing mass that could be calcified/ necrotic/haemorrhagic	Surgical	145	9	0	7
Millet 2011	Secondary or tertiary hospital, France	Retrospective	Patients with <4 cm enhancing renal mass undergoing CT‐guided biopsy	96	99	44 (46)	65	2.3	T1a	CECT	‘Washout’ or ‘plateau’ enhancement pattern	Surgical/biopsy	54	10	20	15
Nassiri 2022	University hospital, USA	Prospective	Patients undergoing surgery for renal mass <4 cm	390	390	NR	NR	NR	T1a	CECT	Point maximising product of sensitivity and specificity on ROC curve for a model including clinical and radiomic features	Surgical	203	27	81	79
Nishikawa 2015	University hospital, Japan	Retrospective	Patients with renal masses <4 cm who underwent partial nephrectomy	144	144	49 (34)	61	2.4	T1a	CECT	Early staining in the arterial phase and washout in the late phase	Surgical	95	7	29	13
Takebayashi 1999	Secondary or tertiary hospital, Japan	Retrospective	Haemodialysis patients with suspected renal cancer who underwent nephrectomy	23	222	6 (26)	46	NR	T1	CECT	Renal mass with >10 HU enhancement but without fat density	Surgical	23	2	1	196
Lei 2012	Secondary or tertiary hospital, China	Retrospective	Patients with renal tumours <3 cm who had CECT and CEUS	132	132	59 (45)	45	2.3	T1a	CECT	Unclear	Surgical/biopsy	102	7	17	6
										CEUS			114	7	5	6
Atri 2015	University hospital, Canada	Prospective	Adults with a <4 cm renal mass and biopsy/surgery diagnosis	91	94	35 (38)	62	2.7	T1a	CEUS	Hypovascularity relative to the adjacent cortex in the arterial phase	Surgical/biopsy	24	0	44	26
Elbanna 2021	University hospital, Canada	Retrospective	Patients with solid renal lesions who had CEUS and pathology diagnosis. Those with a definite solid enhancing lesion on CT/MR were excluded	158	161	57 (36)	59	2.3	T1a	CEUS	Arterial‐phase enhancement less than renal cortex	Surgical/biopsy	54	1	68	38
Fu 2013	University hospital, China	Unclear	Patients with a pathology‐proven renal lesion	35	35	13 (37)	45	3.5	T1	CEUS	Shear wave velocity >/= 2.355 m/s	Surgical/biopsy	21	3	4	8
Rowe 2013 (conference abstract)	Secondary or tertiary hospital, Canada	Unclear	Patients with solid renal masses undergoing surgery	31	32	14 (45)	65	3.1	T1	CEUS	Heterogeneous enhancement	Surgical	15	1	9	7
Eisenbrey 2015	University hospital, USA	Prospective	Patients undergoing percutaneous cryoablation of an enhancing solid renal mass	13	13	5 (38)	71	NR	T1a	CEUS	Early contrast washout	Biopsy	8	1	1	3
Sun 2020	University hospital, China	Retrospective	Patients with renal tumours <3 cm	37	37	NR	47	2.4	T1a	CEUS	Not clear	Not stated	22	1	0	14
Sistani 2020	Secondary or tertiary hospital, Canada	Retrospective	Patients who required further characterisation of a renal mass prior to treatment (at the discretion of the treating urologist)	29	30	6 (21)	60	3	T1	[^99m^Tc]Tc‐MIBI	Absence of radiotracer uptake	Surgical/biopsy	21	0	2	7
Tzortzakakis 2017	University hospital, Sweden	Prospective	Patients with solid T1 renal tumours eligible for surgery or biopsy with any renal function	24	31	NR	NR	NR	T1	[^99m^Tc]Tc‐MIBI SPECT/CT	Absence of radiotracer uptake	Surgical/biopsy	13	2	4	12
Viswambaram 2022	University hospital, Australia	Prospective	Adult patients with solid small renal tumours ≥2 cm, or <2 cm if exophytic	70	70	23 (33)	63	3.4	T1	[^99m^Tc]Tc‐MIBI SPECT/CT	No significant tracer uptake considered malignant	Surgical/biopsy	49	4	6	11
Asi 2020	University hospital, Turkey	Prospective	Adults with T1 renal masses undergoing surgery	90	90	23 (26)	55	4	T1	[^99m^Tc]Tc‐MIBI SPECT/CT	Negative uptake	Surgical	64	6	8	12
Gorin 2016	University hospital, USA	Prospective	Patients presenting for surgery for a solid, solitary T1 renal mass not concerning for a metastasis from alternate primary. eGFR >45 ml/min per 1.73 m^2^	50	50	13 (26)	62	3	T1	[^99m^Tc]Tc‐MIBI SPECT/CT	Radiotracer uptake	Surgical	39	2	4	5
Karyagar 2014	University hospital, Turkey	Retrospective	Patients with suspected renal cancer who underwent FDG PET in the 2 weeks prior to surgery	15	15	NR	55	4	T1	[^18^F]FDG PET	Obvious FDG uptake greater than renal parenchyma	Surgical	1	0	12	2
Kumar 2005	Not reported	Retrospective	Patient with renal masses who underwent FDG PET. Glucose level <140 mg/dl	4	4	2 (50)	67	3.2	T1	[^18^F]FDG PET	Positive if FDG uptake was localised and its intensity was greater than the surrounding normal renal parenchyma	Not stated	2	0	1	1
Ozulker 2011	University hospital, Turkey	Prospective	Patients with primary renal masses detected on CT/MR/US undergoing surgery	13	13	8 (62)	56	4.1	T1	[^18^F]FDG PET	FDG uptake greater than renal parenchyma, distinct from the collecting system	Surgical	3	1	8	1
Ramdave 2001	Secondary or tertiary Hospital, Australia	Prospective	Patients with suspected localised primary renal cancer and tumour <7 cm and diagnostic pathology	11	11	5 (45)	68	4.5	T1	[^18^F]FDG PET	Increased intensity of FDG uptake in the lesion relative to comparable normal tissue	Surgical	8	2	0	1
Muselaers 2013	University hospital, Netherlands	Prospective	Patients with incidental renal masses	8	9	6 (75)	63	4.8	T1	[^111^In]‐girentuximab SPECT/CT	Preferential uptake relative to renal parenchyma	Surgical	7	0	0	2
De Silva 2022	University hospital, Australia	Retrospective	Patients with solid renal tumours and diagnostic pathology	64	72	37 (58)	66	4.1	T1	mpMRI	De Silva St George classification scheme (figure 1 in cited manuscript)	Surgical/biopsy	49	3	2	18
Johnson 2019	University hospital, USA	Retrospective	Patient with cT1a renal tumour and mpMRI reporting ccLS and diagnostic pathology	57	63	19 (33)	62	2.7	T1a	mpMRI	ccLS 4‐5	Surgical/biopsy	34	3	15	4
Kay 2018	University hospital, USA	Retrospective	Patients who underwent surgery for renal mass <4 cm with pre‐operative mpMRI	99	99	47 (47)	56	2.2	T1a	mpMRI	A diagnostic algorithm (figure 10 in cited manuscript)	Surgical	68	6	24	6
Golan 2021	Secondary or tertiary hospital, Israel	Prospective	Patients referred with a clinical stage 1 enhancing renal mass	27	29	8 (30)	66	3.7	T1	PSMA PET	All 68Ga‐PSMA‐11‐avid foci with higher uptake than adjacent renal parenchyma	Surgical/biopsy	15	2	9	3

Abbreviations: [^18^F]FDG PET, [^18^F]fluorodeoxyglucose positron emission tomography; [^99m^Tc]Tc‐MIBI SPECT/CT, [^99m^Tc]Tc‐sestamibi single‐photon emission computed tomography/computed tomography; CECT, contrast‐enhanced computed tomography; CEUS, contrast‐enhanced ultrasound; mpMRI, multiparametric magnetic resonance imaging; NR, not reported; PSMA PET, [^68^Ga]Ga‐prostate‐specific membrane antigen‐11 positron emission tomography.

Participant demographics were as follows: mean age 59 years, 63% male, mean lesion size 3.2 cm, prevalence of renal malignancy 69% (IQR 50%–78%). For comparison, population level age‐specific incidence of kidney cancer is highest in >65 year olds, and 62% of kidney cancer cases occur in men.^13^ Participant ethnicity was reported in three studies, all from the United States as follows: White participants 56%–84%, Black participants 6%–38%, Asian 6%–7% and Hispanic up to 16%.^14^, ^15^, ^16^ For comparison, US census data reports population‐level ethnicity to be 76% White, 14% Black, 6% Asian and 4% mixed/other.^17^ Hispanic origin, considered a distinct concept to race, is 19% (of any race).^17^

The target condition was defined as any malignant lesion in 20 included studies,^16^, ^18^, ^19^, ^20^, ^21^, ^22^, ^23^, ^24^, ^25^, ^26^, ^27^, ^28^, ^29^, ^30^, ^31^, ^32^, ^33^, ^34^, ^35^, ^36^ and we were able to deduce diagnostic accuracy measures for the target condition in the remaining seven studies from the reported data, despite it not being the target condition.^14^, ^15^, ^37^, ^38^, ^39^, ^40^, ^41^

Eight studies received non‐industry funding,^15^, ^18^, ^22^, ^27^, ^28^, ^33^, ^35^, ^38^ three studies were funded or part funded by industry,^14^, ^19^, ^41^ 11 studies did not report the source of funding,^23^, ^25^, ^26^, ^29^, ^30^, ^31^, ^32^, ^36^, ^37^, ^39^, ^40^ and five stated no funding was received.^16^, ^20^, ^21^, ^24^, ^34^

### 3.3 Risk of bias and applicability

**FIGURE 2:**
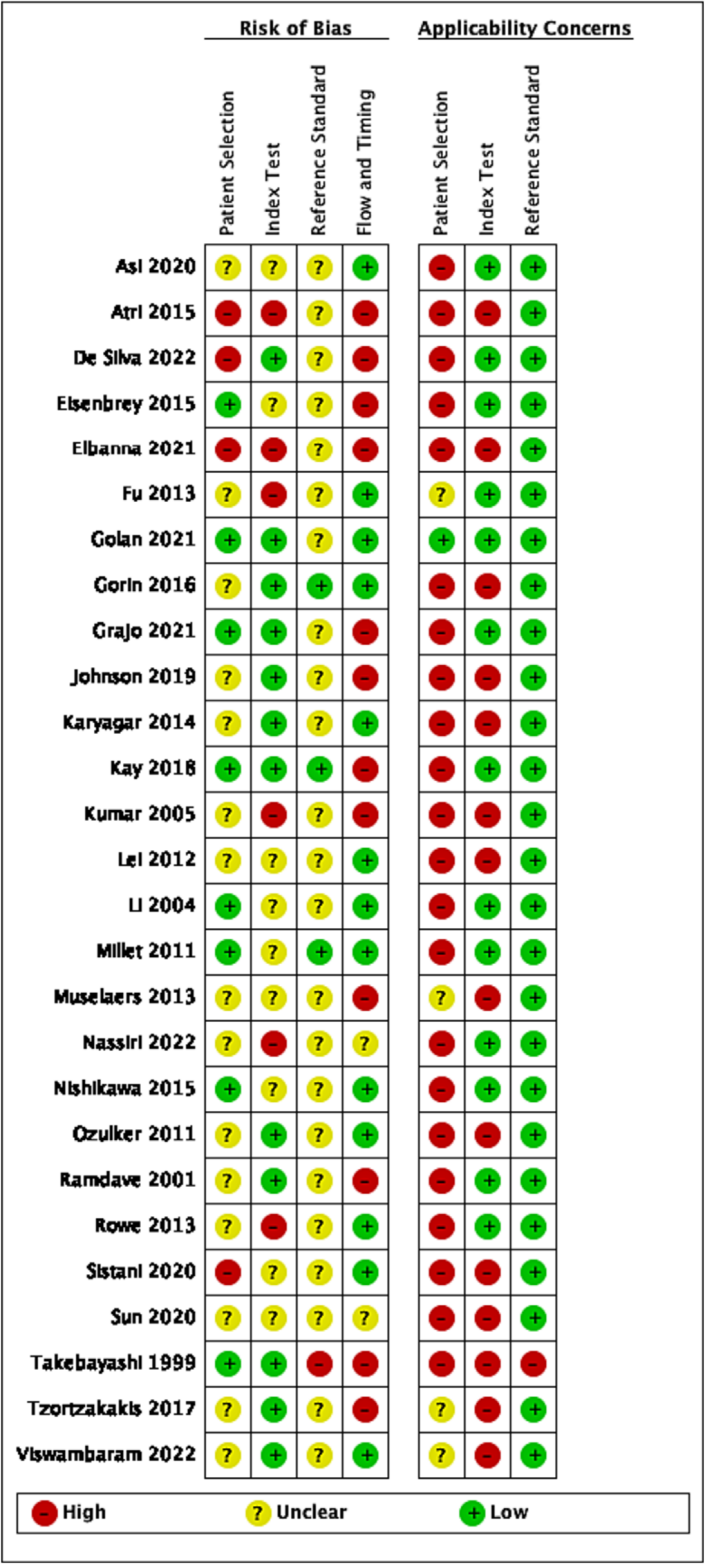
Risk of bias and applicability concerns summary: review authors' judgement about each domain for each included study.

Overall, there was a high or uncertain risk of bias for at least one domain in all included studies (Figure 2).

Eleven included studies were prospective,^14^, ^15^, ^16^, ^18^, ^19^, ^23^, ^32^, ^33^, ^36^, ^38^, ^41^ 14 were retrospective,^20^, ^21^, ^24^, ^25^, ^26^, ^27^, ^28^, ^29^, ^30^, ^31^, ^35^, ^37^, ^39^, ^40^ and two studies were not clear.^22^, ^34^ All studies were single centre. There was one fully paired retrospective comparative study of CEUS versus CECT,^28^ and all others were cross sectional diagnostic accuracy studies of a single index test.

#### 3.3.1 Participant selection

Patient selection was heterogeneous across studies, with the majority of participants included based on management strategy, including partial nephrectomy,^31^ nephrectomy,^40^ any surgical resection,^15^, ^16^, ^18^, ^24^, ^25^, ^26^, ^29^, ^32^, ^34^ ablation^14^ or patients who underwent CT guided biopsy.^30^ Other patients were selected on the basis of having a histological diagnosis from any of the following as part of standard clinical care: biopsy, fine needle aspiration or surgery.^19^, ^20^, ^22^, ^27^, ^28^, ^32^, ^33^, ^36^, ^37^, ^38^, ^39^ One study included patients referred for CEUS when CT, MR or US was indeterminate.^21^ Two small studies included all‐comers,^23^, ^41^ and in one study, the criteria for case selection were unclear.^35^

We considered surgical‐only populations to have high applicability concerns. Surgical patients are likely to be younger and fitter than surveillance populations,^42^ reflected in the study population of this review being younger on average than population‐level data for kidney cancer. Younger patients are more likely to have benign tumours,^43^ reflected in the high proportion of benign tumours in this review, and cause applicability concerns for the wider population of patients with renal masses.

Thirteen studies restricted eligibility to patients with solid tumours,^14^, ^15^, ^19^, ^20^, ^21^, ^24^, ^29^, ^30^, ^31^, ^34^, ^36^, ^38^, ^41^ seven included both solid and cystic tumours,^18^, ^26^, ^27^, ^32^, ^39^, ^40^ and eight did not report if included lesions were solid, cystic or a mixture.^16^, ^22^, ^23^, ^25^, ^28^, ^33^, ^35^, ^37^

#### 3.3.2 Index test

Criteria for a positive CEUS and MRI tests were at different thresholds in each study, or the threshold was not reported. For CECT, contrast enhancement was generally included in the description of a positive test, with^40^ or without^29^, ^30^, ^31^ a defined increase in Hounsfield units between pre and post contrast phases. Alternative criteria were also described^16^, ^24^ or the threshold not defined.^28^

All five studies reporting diagnostic accuracy of [^99m^Tc]Tc‐sestamibi SPECT/CT used the same threshold of absent radiotracer uptake in the tumour to signify malignancy.^15^, ^18^, ^36^, ^38^, ^39^ [^99m^Tc]Tc‐sestamibi SPECT/CT images were reported by two clinicians in collaboration to reach consensus, limiting the applicability to clinical practice where most diagnostic imaging is reported by a single clinician.

Four small studies, each with 4–15 participants reported the diagnostic accuracy of [^18^F]FDG PET^25^, ^27^, ^32^, ^33^ with a common positive threshold of FDG uptake in the tumour greater than the surrounding renal parenchyma.

#### 3.3.3 Reference standard

Generally, there was poor reporting of reference standard conduct and therefore unclear risk of bias. However, where histology was performed as part of standard care, we deemed applicability concerns to be low in all but one study that described pathologic diagnosis made solely on morphology,^40^ when the addition of immunohistochemistry is a minimum standard. Diagnostic criteria used to identify the target condition were not reported for 23 studies,^14^, ^16^, ^18^, ^19^, ^20^, ^21^, ^22^, ^23^, ^24^, ^25^, ^27^, ^28^, ^29^, ^32^, ^33^, ^34^, ^35^, ^36^, ^38^, ^39^, ^40^, ^41^ one study reported International Society of Urological Pathology guidelines,^15^ and four studies reported the World Health Organisation classification system 2004^26^, ^30^, ^31^ and 2016 editions.^37^ Ten studies stated that the reference test was interpreted without knowledge of the results of the index test.^15^, ^16^, ^19^, ^20^, ^21^, ^22^, ^24^, ^25^, ^26^, ^30^

#### 3.3.4 Flow and timing

Studies were deemed at high risk of bias if some participants were excluded from the analysis.^14^, ^20^, ^21^, ^24^, ^26^, ^27^, ^33^, ^37^, ^38^, ^40^, ^41^

#### 3.3.5 Risk of bias in the comparison

For the single study that included a direct comparison of CEUS versus CECT,^28^ risk of bias in the comparison was unclear for patient selection, conduct or interpretation of the index test, conduct or interpretation of the reference standard and at low risk of bias in the comparison for flow and timing.

### 3.4 Results of individual imaging modalities

**FIGURE 3:**
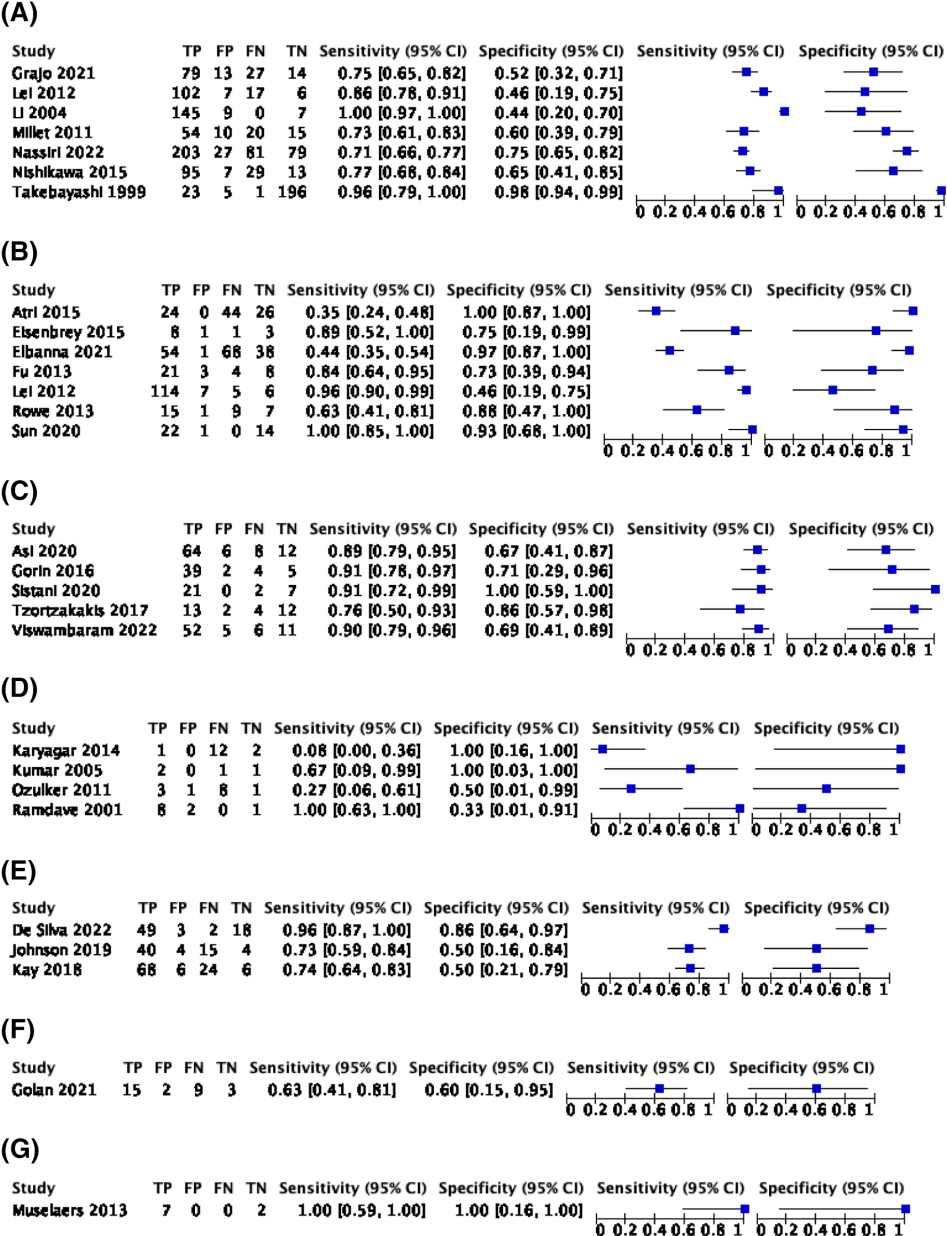
Forest plot of estimates of sensitivity and specificity of (A) contrast‐enhanced computed tomography, (B) contrast‐enhanced ultrasound, (C) [^99m^Tc]Tc‐sestamibi SPECT/CT, (D) [^18^F]FDG PE, (E) multiparametric magnetic resonance imaging, (F) [^68^Ga]Ga‐PSMA‐11 PET and (G) [^111^In]In‐girentuximab SPECT/CT for the diagnosis of tumour malignancy. CI, confidence interval; FN, false negatives; FP, false positives; TN, true negatives; TP, true positives.

Forest plots of estimates of sensitivity and specificity along with the 95% confidence intervals for each included study are presented in Figure 3.

#### 3.4.1 CECT

Seven studies including 1118 patients with 1320 renal lesions reported estimates of sensitivity and specificity for CEUS to detect malignancy in T1 renal tumours ranging from 71% to 100% and 44% to 98%, respectively (Figure 3A). One study was an outlier in forest plots and ROC space,^40^ likely due to the study population of 23 participants with end‐stage renal failure with 222 renal lesions, mostly uncomplicated renal cysts, thus overestimating measures of diagnostic accuracy. Another study reported diagnostic accuracy of a model including clinical and radiomic data (i.e. artificial intelligence‐guided data characterisation) from CT and was therefore not comparable.^16^ The remaining studies used different thresholds to define a positive test, so meta‐analysis was not performed.^12^

#### 3.4.2 CEUS

Seven studies including 197 patients with 504 renal lesions reported estimates of sensitivity and specificity for CEUS to detect malignancy in T1 renal tumours ranging from 35% to 100% and 0% to 100% (Figure 3B). These studies used different thresholds to define a positive test, so meta‐analysis was not performed.^12^

#### 3.4.3 [^99m^Tc]Tc‐sestamibi SPECT/CT

**FIGURE 4:**
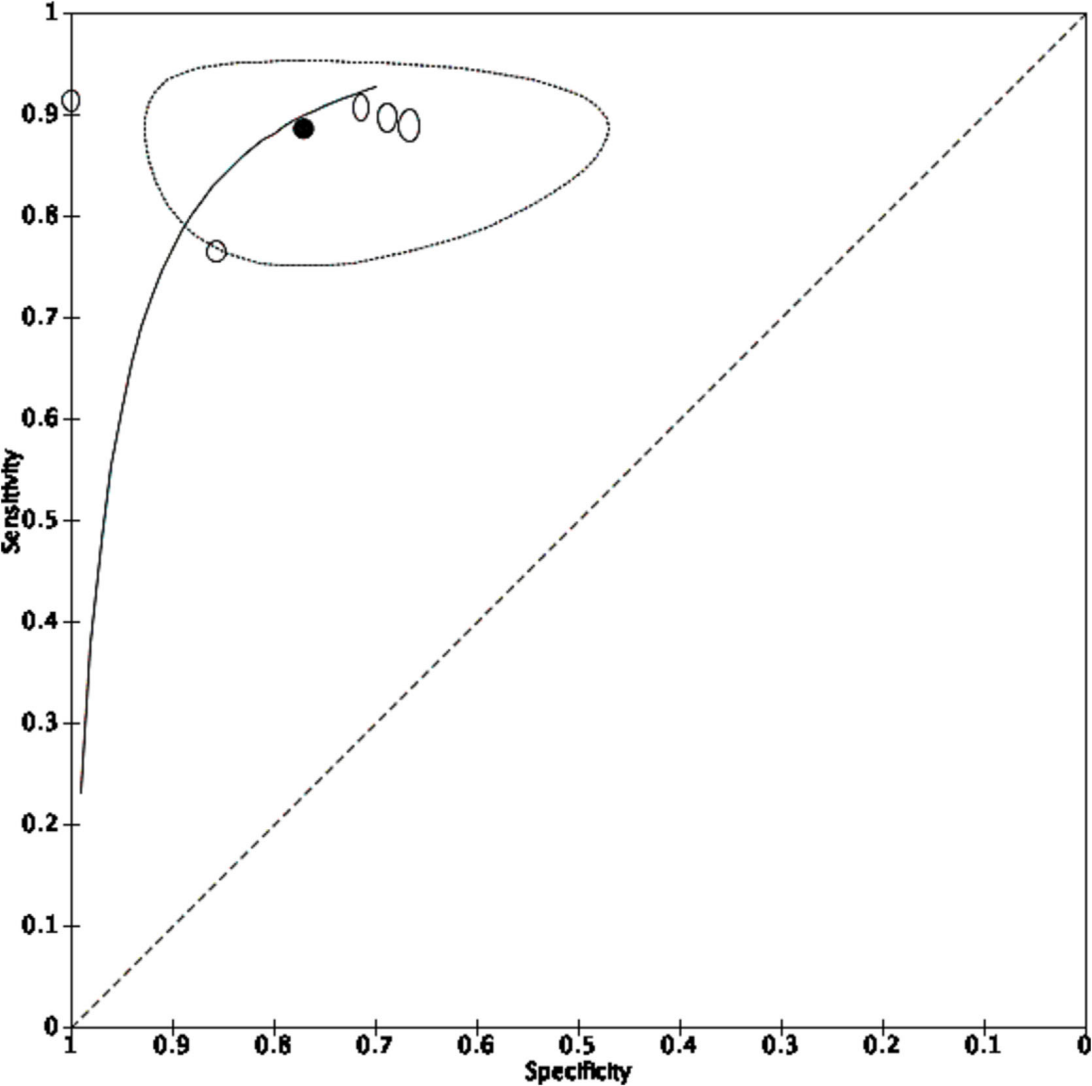
Summary receiver operating characteristic curve of five included studies reporting the diagnostic accuracy of [^99m^Tc]Tc‐sestamibi SPECT/CT to detect malignancy in patients presenting with T1 renal tumours. ○ = estimate from individual study ● = summary estimate = 95% confidence region. Summary estimates of sensitivity and specificity to detect cancer are 88.6% (95% CI 82.7%–92.6%) and 77.0% (95% CI 63.0%–86.9%), respectively.

Five studies including 271 renal lesions in 263 patients reported estimates of sensitivity and specificity for [^99m^Tc]Tc‐sestamibi SPECT/CT to detect malignancy in T1 renal tumours (Figure 3C). All included studies reported measures of diagnostic accuracy at the same positive threshold that was radiotracer uptake in the tumour less than the surrounding renal parenchyma. Meta‐analysis using a univariate fixed‐effect regression model because of sparse data and determined by best model fit returned summary estimates of sensitivity and specificity for [^99m^Tc]Tc‐sestamibi SPECT/CT to detect malignancy of 88.6% (95% CI 82.7%–92.6%) and 77.0% (95% CI 63.0%–86.9%), respectively (Figure 4).

#### 3.4.4 [^18^F]FDG PET

**FIGURE 5:**
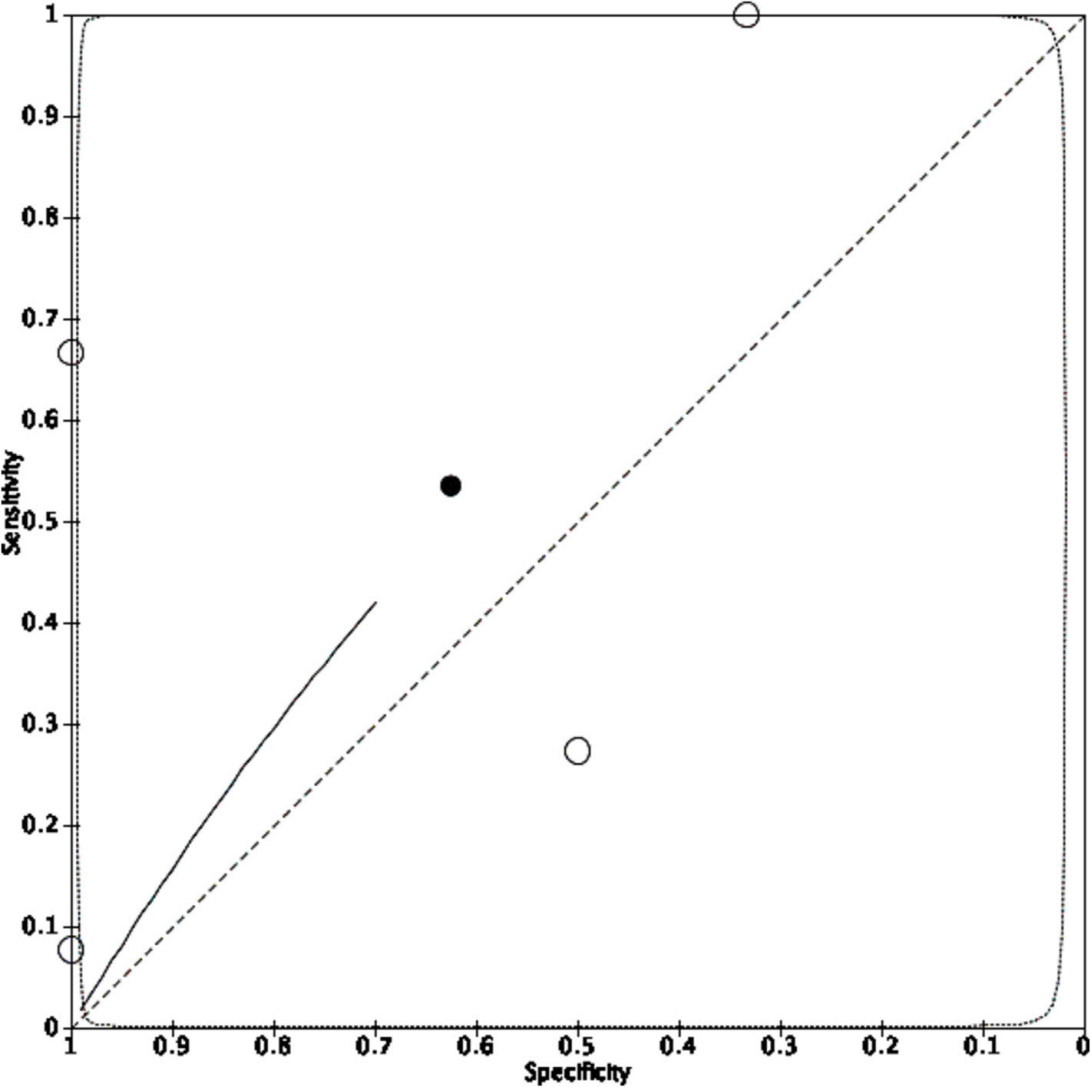
Summary receiver operating characteristic curve of four included studies reporting the diagnostic accuracy of [^18^F]FDG PET to detect malignancy in patients presenting with T1 renal tumours. ○ = estimate from individual study ● = summary estimate = 95% confidence region. Summary estimates of sensitivity and specificity for [^18^F]FDG PET to detect cancer are 53.5% (95% CI 1.6%–98.8%) and 62.5% (95% CI 14.0%–94.5%), respectively.

Four studies including 43 patients with 43 lesions reported estimates of sensitivity and specificity for [18F]FDG PET/CT to detect malignancy in T1 renal tumours (Figure 3D). All included studies reported measures of diagnostic accuracy at the same positive threshold of radiotracer uptake in the tumour relative to the surrounding renal parenchyma. Meta‐analysis using univariate mixed‐effects regression model because of sparse data and determined by best model fit returned summary estimates of sensitivity and specificity for [^18^F]FDG PET to detect malignancy of 53.5% (95% CI 1.6%–98.8%) and 62.5% (95% CI 14.0%–94.5%), respectively (Figure 5). An HSROC model to compare diagnostic accuracy of [18F]FDG PET/CT with [^99m^Tc]Tc‐sestamibi SPECT/CT was attempted but did not converge.

#### 3.4.5 Multiparametric magnetic resonance imaging

Three studies including 220 patients with 234 renal lesions reported estimates of sensitivity and specificity for MRI to detect malignancy in T1 renal tumours ranging from 73% to 96% and 50% to 86%, respectively (Figure 3E). Different thresholds were used to define a positive test, so meta‐analysis was not performed.^12^

#### 3.4.6 [^68^Ga]Ga‐PSMA‐11 PET

One study including 27 patients with 29 renal lesions reported estimates of sensitivity and specificity for [^68^Ga]Ga‐PSMA‐11 PET to detect malignancy in T1 renal tumours of 63% (95% CI 41%–81%) and 60% (95% CI 15%–95%), respectively (Figure 3F).

#### 3.4.7 [^111^In]In‐girentuximab SPECT/CT

One study including eight patients with nine renal lesions reported estimates of sensitivity and specificity for [^111^In]ln‐girentuximab SPECT/CT to detect malignancy in T1 renal tumours of 100% (95% CI 59%–100%) and 100% (95% CI 16%–100%), respectively (Figure 3G).

## 4 DISCUSSION

### 4.1 Principal findings

This is the first systematic review and meta‐analysis of all imaging modalities for the detection of malignancy in T1 renal tumours. We included 27 studies involving 2277 tumours in 2044 participants evaluating the diagnostic accuracy of CECT, CEUS, [^99m^Tc]Tc‐sestamibi SPECT/CT, mpMRI, [18F]FDG PET, [^68^Ga]Ga‐PSMA‐11 PET and [^111^In]ln‐girentuximab SPECT/CT.

Meta‐analysis of studies evaluating [^99m^Tc]Tc‐sestamibi SPECT/CT showed summary estimates of sensitivity and specificity to detect malignancy of 88.6% (95% CI 82.7–92.6) and 77.0% (95% CI 63.0%–86.9%) respectively. Four small, statistically heterogeneous studies evaluating [^18^F]FDG PET had summary estimates of sensitivity and specificity of 53.5% (95% CI 1.6‐98.8%) and 62.5% (95% CI 14.0‐94.5%).

Meta‐analyses for CECT, CEUS and MRI were not appropriate because studies adopted different thresholds to define a positive test and it is not clinically meaningful. The single study that directly compared CEUS with CECT was not of sufficiently high methodological quality to warrant further discussion of the results. The field would benefit from the reporting of diagnostic accuracy at standardised, pre‐specified thresholds, which could be guided by existing literature.

### 4.2 Findings in the context of existing evidence

Previous meta‐analyses have reported diagnostic performance of CEUS versus CECT and/or MRI for renal tumours.^44^, ^45^ In the event of different positive thresholds across studies, an HSROC model is recommended to produce a summary curve rather than point estimates for sensitivity and specificity,^7^ which was not the statistical approach adopted in either review.^44^, ^45^ Further, these reviews chose to include imaging follow‐up as a reference standard. While a period of initial surveillance provides helpful information on the trajectory of a renal lesion, growth rate does not differentiate benign from malignant disease as many cancers remain stable in size^46^ and benign tumours can exhibit growth.^47^ In our own review, we excluded studies where the reference test included imaging surveillance.

There have been two previously published systematic reviews of [^99m^Tc]Tc‐sestamibi SPECT/CT by Wilson et al. in 2020 and Basile et al. in 2023.^48^, ^49^ These reviews reported higher estimates of sensitivity (90%–91%) and specificity (86%) than our own, albeit with overlapping confidence intervals. Several new studies have been published since the former, and the latter is limited by inclusion of case–control studies that are at high risk of bias, excluding histological subtypes other than RCC, oncocytoma or angiomyolipoma and classifying hybrid oncocytic/chromophobe tumours (HOCT) as benign. While misclassifying HOCT as benign is likely of little clinical consequence given their indolent nature, the World Health Organisation defines them as malignant and has recently included them in the emerging entity of ‘low‐grade oncocytic tumours’ (LOT).^50^ Furthermore, both reviews differed from our own by including tumours of all T stages and therefore had a higher proportion of malignant histology (78%–83% vs. 69%). The ability to differentiate benign from malignant tumours is most relevant in the T1 setting where clinicians report higher willingness to manage benign tumours conservatively.^51^ Further prospective studies of [^99m^Tc]Tc‐sestamibi SPECT/CT are awaited,^52^ and work evaluating its role as an replacement test for biopsy, add‐on test, or triage test is needed.

A MRI‐based ‘clear cell likelihood score’ has shown pooled estimates of sensitivity and specificity of 80% (95% CI 75%–85%) and 74% (95% CI 65%–81%) to detect clear cell RCC in a systematic review and meta‐analysis of six studies including 825 T1a renal masses.^53^ Additionally, [^89^Zr]DFO‐girentuximab PET/CT has been reported in a conference abstract to have sensitivity of 86% [80%, 90%] and 87% [79%, 92%], also for detecting clear cell RCC with the full manuscript awaited.^54^ These studies were not included in our review as it was not possible to extract diagnostic accuracy data for benign versus malignant lesions. Clinically, these tests may have a triage role supporting active treatment for patients with a positive test for clear cell RCC; however, patients with a negative test would still require further diagnostics.

Radiomics has received growing interest, including in the setting of renal tumours.^5^, ^55^ Advanced computing may allow extraction of quantitative spatial information from medical imaging to detect differences imperceptible to the human eye. Only one manuscript including radiomics from CT was of sufficient quality for inclusion in this review and reported area under the curve of 0.77 (95% CI 0.69–0.85) for a model including radiomics and clinical factors.^16^ No comparison was made with radiologist reporting of imaging.

### 4.3 Limitations

We applied diagnostic filters in our search strategy to limit the returned texts to a feasible number to screen. The filters used have a sensitivity of 98.6% for MEDLINE^9^ and 100% for Embase,^8^ so the risk of having omitted relevant studies is low.

A limitation of our review is that most participants underwent surgical resection or diagnostic biopsy, due to our inclusion criteria necessitating a histopathological reference standard. In doing so, we limit the applicability of our results to patients on surveillance without histopathological diagnosis.

Eighty‐four studies were excluded from our review because they included all stages of renal tumour and it was not possible to extract diagnostic accuracy data for T1 tumours alone. We advocate future diagnostic accuracy studies reporting measures of diagnostic accuracy for each tumour stage to facilitate future reviews.

We chose per‐lesion rather than per‐participant analysis as information at the level of the lesion is important for clinical decision making. For example, if a patient had multiple synchronous renal lesions—some malignant and others benign—then urologists would favour treating the malignant tumours, and not the benign ones in an effort to preserve renal function. However, this approach assumes independence of the lesions in a single participant, and therefore, measures of diagnostic accuracy are likely overestimated for studies that included participants with multiple lesions.^56^

### 4.4 Deviations from the protocol

We revised our original protocol from including only T1a to all T1 renal tumours due to sparse data for T1a lesions alone. The protocol change was registered with PROSPERO. T1a renal tumours have the highest prevalence of benign histology when compared to tumours of greater size and T stage,^43^ and extended eligibility to larger tumours has likely resulted in a higher prevalence of malignant histology, although the mean size of included tumours was 3.2 cm. For all imaging modalities, it is conceivable that the diagnostic accuracy increases with increasing tumour size due to both resolution limits and less signal contamination in the tumour volume from normal surrounding renal parenchyma.

## 5 CONCLUSIONS

Imaging‐based diagnostics for risk stratifying renal tumours is an unmet need. Currently, the optimal imaging strategy to characterise T1 renal tumours is not clear because of heterogeneity and sparse data as well as a lack of direct comparisons. [^99m^Tc]Tc‐sestamibi SPECT/CT is an emerging tool, but further studies are required to inform its role in clinical practice. We advocate future diagnostic accuracy studies reporting performance at each tumour stage and standardisation of the diagnostic threshold used to consider CT, MRI and CEUS positive for cancer.

## AUTHOR CONTRIBUTIONS

The study was conceived and designed by HW, ME, MGBT and KG. The protocol was developed and published (HW, JBF, VWSC, RH, VK, ME, MGBT, KG). Study screening and data extraction was performed by HW, JBF, VM, PI, VWSC and EZ. Analysis was performed by HW with support from KG. The manuscript was written by HW, with revisions from JBF, RH, VK, MGBT and KG. All co‐authors approved the final version of the manuscript.

## CONFLICT OF INTEREST STATEMENT

HW receives salary support from The Urology Foundation, Pan London Cancer Alliance (Royal Marsden Partners, North Central London Cancer Alliance, North East London Cancer Alliance, South East London Cancer Alliance and the NIHR BRCs) and the Wellcome/EPSRC Centre for Interventional and Surgical Sciences. The promotions and salaries of KG are dependent upon the publishing of research protocols and findings. Other authors have no relevant interests to declare.

## Supporting information


**Appendix S1.** Search Strategies.

**Appendix S2.** Customised QUADAS‐2 and QUADAS‐C tools.

**Appendix S3.** Statistical code.

**Data S1.** Supporting Information.
