# Supplementary material for: Imaging modalities for characterising T1 renal tumours: A systematic review and meta‐analysis of diagnostic accuracy
Source: BJUI Compass. 2024 Jun 21;5(7):636–50. doi: 10.1002/bco2.355 (PMC11249832; doi:10.1002/bco2.355)
Supplement: Supplementary file 1 — Appendix S1. Search Strategies. [file BCO2-5-636-s003.docx]

Appendix 1

**Medline Search Strategy**

1. exp Kidney Neoplasms/
2. ((renal or kidney) adj3 (tumor or tumors or tumour or tumours or cancer or cancers or carcinoma or carcinomas or mass or masses or neoplasm or neoplasms or lesion or lesions or malignancy or malignancies or adenocarcinoma or adenocarcinomas)).tw,kw.
3. (((tumour or tumor) adj grawitz) or hypernephroma).tw,kw.
4. exp diagnostic imaging/
5. (CT or PET or MRI or US or CEUS or SPECT or "SPECT/CT" or scintigraphy or ((computed or computerised or computerized or magneti* or MR or proton or postiron or emission or ultrasound) and (tomogra* or imaging or scan or scans))).tw,kw.
6. sensitiv:.mp. OR diagnos:.mp. OR di.fs.
7. (histology or pathology or histopathology or biopsy).tw,kw.
8. 1 or 2 or 3
9. 4 or 5
10. 6 and 7 and 8 and 9
11. 6 and 8 and 9
12. 7 and 8 and 9
13. 8 and 9

**Embase Search Strategy**

1. exp kidney tumor/
2. ((renal or kidney) adj3 (tumor or tumors or tumour or tumours or cancer or cancers or carcinoma or carcinomas or mass or masses or neoplasm or neoplasms or lesion or lesions or malignancy or malignancies or adenocarcinoma or adenocarcinomas)).tw,kw.
3. (((tumour or tumor) adj grawitz) or hypernephroma).tw,kw.
4. exp diagnostic imaging/
5. (CT or PET or MRI or US or CEUS or SPECT or "SPECT/CT" or scintigraphy or ((computed or computerised or computerized or magneti* or MR or proton or postiron or emission or ultrasound) and (tomogra* or imaging or scan or scans))).tw,kw.
6. (histology or pathology or histopathology or biopsy).tw,kw
7. di.fs. or predict.tw. or specificity.tw.
8. 1 or 2 or 3
9. 4 or 5
10. 6 and 7 and 8 and 9
11. 7 and 8 and 9
12. 6 and 8 and 9
13. 8 and 9

**Science Citation Index (Web of Science) search strategy**

#1 TS=(kidney OR renal)

#2 TS=(tumour OR tumor OR tumours OR tumors OR cancer OR cancers OR carcinoma OR carcinomas OR mass OR masses OR neoplasm OR neoplasms OR lesion OR lesions)

#3 TS=(CT OR PET OR MRI OR US OR CEUS OR SPECT OR SPECT/CT OR scintigraphy OR ((computed OR computerised OR computerized OR magneti* OR MR OR proton OR positron OR emission OR ultrasound) AND (tomogra* OR imaging OR scan OR scans)))

#4 TS=(pathology OR histology OR histopathology OR biopsy)

#5 TS=(sensitiv* OR “predictive value” or diagnostic accuracy)
#6 #1 AND #2 AND #3 AND #4 AND #5

**The Cochrane Library search strategy**

#1 (MeSH descriptor: [Kidney Neoplasms] explode all trees

#2 ((kidney OR renal):ti,ab,kw

#3 (tumour OR tumor OR tumours OR tumors OR cancer OR cancers OR carcinoma OR carcinomas OR mass OR masses OR neoplasm OR neoplasms OR lesion OR lesions):ti,ab,kw

#4 #1 OR (#2 AND #3)

#5 MeSH descriptor: [Diagnostic Imaging] explode all trees

#6 (CT OR PET OR MRI OR US OR CEUS OR SPECT OR "SPECT/CT" OR scintigraphy):ti,ab,kw

#7 (computed OR computerised OR computerized OR magneti* OR MR OR proton OR positron OR emission OR ultrasound):ti,ab,kw

#8 (tomogra* OR imaging OR scan OR scans):ti,ab,kw

#9 #5 OR #6 OR (#7 AND #8)

#10 pathology OR histology OR histopathology OR biopsy

#11 #4 AND #9 AND #10

**Clinicaltrials.gov**

((kidney OR renal) AND (tumour OR tumor OR cancer OR carcinoma OR mass OR neoplasm OR lesion)) AND (CT OR PET OR MRI OR US OR CEUS OR SPECT OR SPECT/CT OR computed OR magnetic OR MR OR proton OR positron OR emission OR ultrasound)

**WHO Trials Register**

((kidney OR renal) AND (tumour OR tumor OR cancer OR carcinoma OR mass OR neoplasm OR lesion)) AND (CT OR PET OR MRI OR US OR CEUS OR SPECT OR SPECT/CT OR computed OR magnetic OR MR OR proton OR positron OR emission OR ultrasound)
