# Supplementary material for: Imaging modalities for characterising T1 renal tumours: A systematic review and meta‐analysis of diagnostic accuracy
Source: BJUI Compass. 2024 Jun 21;5(7):636–50. doi: 10.1002/bco2.355 (PMC11249832; doi:10.1002/bco2.355)
Supplement: Supplementary file 2 — Appendix S2. Customised QUADAS‐2 and QUADAS‐C tools. [file BCO2-5-636-s002.docx]

**Customised QUADAS-2 tool**

| **Item** | **Response** |
| --- | --- |
| **PARTICIPANT SELECTION (1) – RISK OF BIAS** |  |
| 1. Was a consecutive or random sample of patients enrolled? | **Yes** – if paper states consecutive or random, or if *all* participants meeting explicit study eligibility criteria within a specified time frame were clearly included  **No** – if paper describes other method of sampling  **Unclear** – if participant sampling not described |
| 1. Was a case-control avoided?   *Note case-control design was an exclusion condition of this review | **Yes** – if case-control design clearly not used  **No** – study identified cases of renal cell carcinoma (RCC) or RCC subtypes as well as benign tumours (oncocytoma, angiomyolipoma) from histopathology and then reviewed imaging findings for these cases  **Unclear** – not clearly described |
| 1. Did the study avoid inappropriate exclusions? (e.g. patients with renal failure, exclusion of very small tumours e.g. <2cm, including only cases that were indeterminate with other imaging techniques). | **Yes** – inappropriate exclusions avoided  **No** – where selection criteria likely led to exclusion of those with easier or more difficult to interpret imaging findings  **Unclear** – where selection criteria are not clearly reported |
| **Could selection of participants have introduced bias?**  If answers to all questions 1) + 2) + 3) = Yes  If answers to **any** question 1) OR 2) OR 3) = No  If above options do not apply | **Risk is Low**  **Risk is High**  **Risk is Unclear** |
| **PARTICIPANT SELECTION (1) – CONCERNS REGARDING APPLICABILITY** |  |
| 1. Are the included patients and chosen study setting appropriate to answer the question, i.e., are the study results generalisable? | **Yes** – included patients are generally representative of all patients who present in the usual practice setting  **No** – study participants restricted to those in sub-groups (for example based on age, renal function, populations undergoing one particular type of treatment, or only solid or cystic tumours were included); study did not perform imaging test for *all* included participants with renal tumours.  **Unclear** – included patients not described |
| **Is there a concern that included participants do not match the review question?**  If answer to question is Yes  If answer to question is No  If answer to question is Unclear | **Risk is Low**  **Risk is High**  **Risk is Unclear** |
| **INDEX TEXT (2) – RISK OF BIAS** |  |
| 1. Was the index test interpreted without knowledge of the results of the reference standard? | **Yes** – images were interpreted and reported before histopathology reference standard was known, or interpretation was blinded to the reference standard result  **No –** images were not interpreted in a blinded fashion  **Unclear** – if blinding or timing of the interpretation of the index test relative to the reference standard is unclear |
| 1. Was there a **pre-specified** (i.e., before study commencement), formal threshold by which malignancy was confirmed for the index imaging modality? | **Yes –** study provides diagnostic threshold of imaging modality to confirm malignancy which was defined **prior to study commencement**  **No** – there was a defined threshold for the imaging modality to confirm malignancy but this was introduced at analysis of results to potentially maximise sensitivity or specificity  **Unclear** – it was not possible to tell if diagnostic threshold was pre-specified |
| **Could the conduct or interpretation of the index test have introduced bias?**  If answer to both questions 1) + 2) = Yes  If answer to either question 1) OR 2) = No  If above options do not apply | **Risk is Low**  **Risk is High**  **Risk is Unclear** |
| **INDEX TEST (2) – CONCERN ABOUT APPLICABILITY** |  |
| 1. Was the test applied and interpreted in a clinically applicable manner? | **Yes** – index imaging test was interpreted by a single observer who was a qualified or trained clinician  **No** – Image acquisition and interpretation was done by: (i) an inadequately trained clinician (ii) by more than one individual (e.g., group consensus)  **Unclear** – study does not report training of individuals or protocols for imaging acquisition/interpretation |
| 1. Were thresholds for diagnosis reported in sufficient detail to allow replication? | **Yes** – criteria for a diagnosis of malignancy on index imaging were clearly reported to enable replication in practice  **No** – criteria for a diagnosis of malignancy on index imaging were not reported in sufficient detail to allow replication  **Unclear** – criteria for a diagnosis of malignancy on index imaging was reported but lack of detail meant it was unclear if it could be adequately replicated |
| **Is there concern that the index test, its conduct, or interpretation differ from the review question?**  If answer to both questions 1) + 2) = Yes  If answer to either question 1) OR 2) = No  If above options do not apply | **Risk is Low**  **Risk is High**  **Risk is Unclear** |
| **REFERENCE STANDARD (3) – RISK OF BIAS** |  |
| 1. Is the reference standard likely to correctly classify the target condition? | **Yes** – study adopted the World Health Organisation/International Society of Urological Pathology definition of cancer for the histopathological reference standard  **No** – study provides alternative definition of malignancy for histopathological reference standard considered to represent a different threshold than that of our review protocol  **Unclear** – study does not provide clear definition of malignancy for the histopathological reference standard |
| 1. Were the reference standard results interpreted without knowledge of the results of the index test? | **Yes –** those interpreting the histopathology were blinded to the imaging test result  **No** – study reports that those interpreting the histopathology were not blinded to the imaging test result **Unclear** – blinding of those interpreting the histopathology reference standard result was not clearly reported |
| **Could the reference standard, its conduct, or its interpretation have introduced bias?**  If answer to both questions 1) + 2) = Yes  If answer to either question 1) OR 2) = No  If above options do not apply | **Risk is Low**  **Risk is High**  **Risk is Unclear** |
| **REFERENCE STANDARD (3) – CONCERN ABOUT APPLICABILITY** |  |
| Was interpretation of the reference standard done by an appropriately trained and experienced clinician? | **Yes** – study reports the use of appropriately trained individuals e.g. uropathologist  **No** – study reports the inclusion of individuals who are not appropriately trained or experienced e.g. resident pathologist without supervision  **Unclear** – study does not report the experience or training of those individuals that are responsible for the delivery of the reference standards |
| **Is there concern that the target condition as defined by the reference standard does not match the review question?**  If answer to question is Yes  If answer to question is No  If answer to question is Unclear | **Risk is Low**  **Risk is High**  **Risk is Unclear** |
| **FLOW AND TIMING (4): RISK OF BIAS** |  |
| 1. Was there an appropriate interval between index test and reference standard? | **Yes** – study reports the index test was performed prior to surgery or biopsy within a short diagnostic window in which the disease is unlikely to have changed/progressed i.e. time between diagnostic imaging and treatment as part of standard clinical care. Alternatively, if a biopsy was performed prior to the index test but with a sufficiently long window for bleeding/swelling as a result of biopsy to have resolved i.e. >6 weeks  **No** – study includes cases where the index test took place a short interval after biopsy e.g. <6 weeks  **Unclear** – study does not report time interval between index text and reference standard |
| 1. Did all participants undergo the histopathological reference standard with the same approach to surgery/biopsy?   *Note eligibility for inclusion in this review was a histopathological reference standard | **Yes** – study reports all participants underwent histopathological assessment with a consistent approach to surgery or biopsy  **No** – study reports that some participants went without histopathological assessment  **Unclear** – study does not clearly describe the approach to selecting the reference standard |
| 1. Were all participants included in the analysis? | **Yes** – no participants were excluded  **No** – study reported exclusion of participants from analysis or loss to follow-up  **Unclear** – study does not clearly describe if all participants were included in the analysis |
| Could the participant flow have introduced bias?  If answers to all questions 1) + 2) + 3) = Yes  If answers to **any** question 1) OR 2) OR 3) = No  If above options do not apply: | **Risk is Low**  **Risk is High**  **Risk is Unclear** |

**Customised QUADAS-C tool**

| **Item** | **Response** |
| --- | --- |
| **PARTICIPANT SELECTION (1) – RISK OF BIAS IN THE COMPARISON** | |
| 1. Was the risk of bias for each index test judged ‘low’ for this domain? | **Yes** - if the risk of bias judgment for single test accuracy was ‘low’ for each index test  **No** - if the risk of bias judgment for single test accuracy was ‘high’ or ‘unclear’ for each index test |
| 2. Was a fully paired or randomised design used? | **Yes** - if one of the following methods was used for allocating patients to index tests: (a) each patient receiving all of the index tests (fully paired design) or (b) random allocation of patients to one of the index tests (randomized design)  **No** – if each participant did not receive all of the index test, and the allocation was not random  **Unclear** – if allocation of tests not clearly reported |
| 3. Was the allocation sequence random? | **Yes** - if the study generated a truly random allocation sequence, for example, computer-generated random numbers and random number tables  **No** – if method other than the above reported  **Unclear** – if the method for allocation was not clearly reported |
| 4. Was the allocation sequence concealed until patients were enrolled and assigned to index tests | **Yes** - if the study used appropriate methods to conceal allocation, such as central randomization schemes and opaque sealed envelopes  **No** – if allocation sequence was not concealed, such as described above  **Unclear** – if the allocation sequence was not clearly reported |
| Could the selection of patients have introduced bias in the comparison?  If answer to both questions 1) + 2) = Yes  If answer to either question 1) OR 2) = No  If above options do not apply | **Low risk**  **High risk**  **Unclear risk** |
| **INDEX TEST (2) – RISK OF BIAS IN THE COMPARISON** | |
| 1. Was the risk of bias for each index test judged ‘low’ for this domain? | **Yes** - if the risk of bias judgment for single test accuracy was ‘low’ for each index test  **No** - if the risk of bias judgment for single test accuracy was ‘high’ or ‘unclear’ for each index test |
| 2. Were the index test results interpreted without knowledge of the results of the other index test | **Yes** - if index test A was interpreted blind to the results of index test B and vice versa, or if blinding is not necessary if none of the index tests involve subjective interpretation  **No** – if interpretation of the index test(s) is subjective and not blinded to one another  **Unclear** – if blinding was not clearly reported |
| 3. Is undergoing one index test unlikely to affect the performance of the other index test | **Yes** - if one index test cannot influence or interfere with the results of subsequently performed index test(s)  **No** – if one index test can influence or interfere with the results of subsequently performed index tests e.g. injection of iodine-based contrast for CT followed by another X-ray based imaging modality  **Unclear** – if it is not clear whether one index test is likely to affect the performance of the other index test |
| 4. Were the index tests conducted and interpreted without advantaging one of the tests | **Yes** - if there were no differences between the index tests that may unfairly benefit one of the tests. Differences between tests that reflect clinical practice are acceptable  **No** - there were differences between the index tests that might unfairly benefit one of the tests e.g. index test A was performed by an expert and index test B by a nonexpert  **Unclear** – it is not clearly reported if index tests were conducted and interpreted without advantaging one of the tests |
| Could the conduct or interpretation of the index tests have introduced bias in the comparison?  If answer to both questions 1) + 2) = Yes  If answer to either question 1) OR 2) = No  If above options do not apply | **Low risk**  **High risk**  **Unclear risk** |
| **REFERENCE STANDARD (3) – RISK OF BIAS IN THE COMPARISON** | |
| 1. Was the risk of bias for each index test judged ‘low’ for this domain? | Yes - if the risk of bias judgment for single test accuracy was ‘low’ for each index test  No - if the risk of bias judgment for single test accuracy was ‘high’ or ‘unclear’ for each index test |
| 2. Did the reference standard avoid incorporating any of the index tests | **Yes** - if none of the index tests were part of the reference standard  **No** – if the index tests formed (part of) the reference standard  **Unclear** – if it was not clearly reported if the index tests were part of the reference standard |
| Could the reference standard, its conduct, or its interpretation have introduced bias in the comparison?]  If answer to both questions 1) + 2) = Yes  If answer to either question 1) OR 2) = No  If above options do not apply | **Low risk**  **High risk**  **Unclear risk** |
| **FLOW AND TIMING (4) – RISK OF BIAS IN THE COMPARISON** | |
| 1. Was the risk of bias for each index test judged ‘low’ for this domain? | **Yes** - if the risk of bias judgment for single test accuracy was ‘low’ for each index test  **No** - if the risk of bias judgment for single test accuracy was ‘high’ or ‘unclear’ for each index test |
| 2. Was there an appropriate interval between the index tests? | **Yes** – index tests were conducted within a short diagnostic window in which the disease is unlikely to have changed/progressed i.e. time from diagnosis to definitive treatment as part of standard care. All index tests were conducted prior to invasive intervention  **No** – index tests were conducted over a time period in which the disease may have changed or progressed >6 months, or biopsy/surgery took place between index test A and B  **Unclear** – the interval between index tests is not clearly reported, or timing of biopsy is not clear |
| 3. Was the same reference standard used for all index tests?  *Note eligibility for inclusion in this review was a histopathological reference standard | **Yes** - if histopathology (from surgery or biopsy) was used in all patients receiving index test A and index test B  **No** – if multiple reference standards were used e.g. histopathology or imaging follow up and these reference standards were not the same for patients receiving index test A and index test B  **Unclear** – it was unclear if the same reference standard were used for all index tests |
| 4. Are the proportions and reasons for missing data similar across index tests? | **Yes** - if there is no missing data, or if the proportion and reasons for missing data are similar for index test A and index test B e.g. data is unavailable, invalid, inconclusive, or if patients are excluded from the analysis  **No** – There is missing data and the proportions or reasons for missing data are not similar for index test A and B  **Unclear** – it is not clearly reported if there is missing data or if the proportions and reasons for missing data are the same across index tests |
| Could the patient flow have introduced bias in the comparison?  If answer to both questions 1) + 2) = Yes  If answer to either question 1) OR 2) = No  If above options do not apply | **Low risk**  **High risk**  **Unclear risk** |

| C3.1: Answer ‘yes’ if the risk of bias judgment for single test accuracy (question 3.3 in QUADAS-2) was ‘low’ for each index test.  C3.2: Answer ‘yes’ if none of the index tests were part of the reference standard. Note that this issue is different from blinding (signaling question 3.2 in QUADAS-2).  C3.3: Risk of bias can be judged ‘low’ if signaling questions C3.1 and C3.2 were answered ‘yes’. If at least one question was answered ‘no’, users should consider a ‘high risk of bias’ judgment if the bias associated with the design feature is of such concern that the entire domain is deemed problematic. |
| --- |
