# Supplementary material for: Imaging modalities for characterising T1 renal tumours: A systematic review and meta‐analysis of diagnostic accuracy
Source: BJUI Compass. 2024 Jun 21;5(7):636–50. doi: 10.1002/bco2.355 (PMC11249832; doi:10.1002/bco2.355)
Supplement: Supplementary file 3 — Appendix S3. Statistical code. [file BCO2-5-636-s001.docx]

**Appendix 3 – Meta-analysis Code**

**[^99m^Tc]Tc-sestamibi SPECT/CT**

"data DiagnosticTestMetaAnalysis;

input Study_id  TP  FP  FN  TN;

datalines;

1  64  6  8  12

2  39  2  4  5

3  21  0  2  7

4  13  2  4  12

5  49  4  6  11

run;

/* Modify the dataset for the bivariate analysis */

data dt;

set  DiagnosticTestMetaAnalysis;

sens=1; spec=0; true=tp; n=tp+fn; output;

sens=0; spec=1; true=tn; n=tn+fp; output;

run;

/* Ensure that both records for a study are clustered together  */

proc sort data=dt;

by study_id ;

run;

/* MODEL 1 */

/* Save NLMIXED output in the following datasets*/

ods output ParameterEstimates=pet1 FitStatistics=fitt1 additionalestimates=addest1

CovMatParmEst=covparmestt1 ConvergenceStatus=convgstatt1;

/* Run the bivariate random effects logistic regression model for sensitivity and specificity */

/* The cov option requests that a covariance matrix is printed for all model parameter estimates.*/

proc nlmixed data=dt cov tech=quanew lis=5;

parms msens=2 mspec=1  s2usens=0 s2uspec=0 covsesp=0;

logitp=(msens+usens)*sens+(mspec+uspec)*spec;

p = exp(logitp)/(1+exp(logitp));

model true ~ binomial(n,p);

random usens uspec ~ normal([0,0],[s2usens,covsesp,s2uspec]) subject=study_id out=randeffs;

estimate 'logLR+' log((exp(msens)/(1+exp(msens)))/(1-(exp(mspec)/(1+exp(mspec)))));

estimate 'logLR-' log((1-(exp(msens)/(1+exp(msens))))/(exp(mspec)/(1+exp(mspec))));

run;

/* Obtain summary sens and spec from the model 1*/

/* change the number if this is for a different model*/

data summary1;

set pet1;

if parameter = 'msens' then name = 'Sensitivity';

else if parameter = 'mspec' then name = 'Specificity';

if parameter = 'msens' or parameter ='mspec' then summary=100 * exp(estimate)/(1 + exp(estimate));

if parameter = 'msens' or parameter ='mspec' then summlower=100 * exp(lower)/(1 + exp(lower));

if parameter = 'msens' or parameter ='mspec' then summupper=100 *exp(upper)/(1 + exp(upper));

output;

run;

/* Obtain summary LR from the model 1 */

data summaryLR1;

set addest1;

summary=exp(estimate);

summlower=exp(lower);

summupper=exp(upper);

output;

run;

PROC EXPORT DATA= WORK.SUMMARY1

OUTFILE = ""N:\Downloads\SASFile\IndeterminatesExcluded\Summary1.csv""

DBMS=CSV REPLACE;

RUN;

/* Export parameter estimates table */

PROC EXPORT DATA= WORK.pet1

OUTFILE = ""N:\Downloads\SASFile\IndeterminatesExcluded\Parameter estimates1.csv""

DBMS=CSV REPLACE;

RUN;

/* Export the summary LR as an Excel .csv file */

PROC EXPORT DATA= WORK.SUMMARYLR1

OUTFILE = ""N:\Downloads\SASFile\IndeterminatesExcluded\SummaryLR1.csv""

DBMS=CSV REPLACE;

RUN;

/* Export Fit statistics table */

PROC EXPORT DATA= WORK.fitt1

OUTFILE = ""N:\Downloads\SASFile\IndeterminatesExcluded\Fit statistics1.csv""

DBMS=CSV REPLACE;

RUN;

/* Export covariance parameter estimates table */

PROC EXPORT DATA= WORK.covparmestt1

OUTFILE = ""N:\Downloads\SASFile\IndeterminatesExcluded\Covariance parameter estimates1.csv""

DBMS=CSV REPLACE;

RUN;

/* MODEL 2 */

ods output ParameterEstimates=pet2 FitStatistics=fitt2 additionalestimates=addest2 CovMatParmEst=covparmestt2 ConvergenceStatus=convgstatt2;

/* Run univariate random effects logistic regression models for sensitivity and specificity, i.e., ignore the correlation */

proc nlmixed data=dt cov tech=quanew lis=5;

parms msens=2 mspec=1  s2usens=0 s2uspec=0 ;

logitp=(msens+usens)*sens+(mspec+uspec)*spec;

p = exp(logitp)/(1+exp(logitp));

model true ~ binomial(n,p);

random usens uspec ~ normal([0,0],[s2usens,0,s2uspec]) subject=study_id out=randeffs;

estimate 'logLR+' log((exp(msens)/(1+exp(msens)))/(1-(exp(mspec)/(1+exp(mspec)))));

estimate 'logLR-' log((1-(exp(msens)/(1+exp(msens))))/(exp(mspec)/(1+exp(mspec))));

run;

/* Obtain summary sens and spec from the model 2*/

/* change the number if this is for a different model*/

data summary2;

set pet2;

if parameter = 'msens' then name = 'Sensitivity';

else if parameter = 'mspec' then name = 'Specificity';

if parameter = 'msens' or parameter ='mspec' then summary=100 * exp(estimate)/(1 + exp(estimate));

if parameter = 'msens' or parameter ='mspec' then summlower=100 * exp(lower)/(1 + exp(lower));

if parameter = 'msens' or parameter ='mspec' then summupper=100 *exp(upper)/(1 + exp(upper));

output;

run;

/* Obtain summary LR from the model 2 */

data summaryLR2;

set addest2;

summary=exp(estimate);

summlower=exp(lower);

summupper=exp(upper);

output;

run;

PROC EXPORT DATA= WORK.SUMMARY2

OUTFILE = ""N:\Downloads\SASFile\IndeterminatesExcluded\Summary2.csv""

DBMS=CSV REPLACE;

RUN;

/* Export parameter estimates table */

PROC EXPORT DATA= WORK.pet2

OUTFILE = ""N:\Downloads\SASFile\IndeterminatesExcluded\Parameter estimates2.csv""

DBMS=CSV REPLACE;

RUN;

/* Export the summary LR as an Excel .csv file */

PROC EXPORT DATA= WORK.SUMMARYLR2

OUTFILE = ""N:\Downloads\SASFile\IndeterminatesExcluded\SummaryLR2.csv""

DBMS=CSV REPLACE;

RUN;

/* Export Fit statistics table */

PROC EXPORT DATA= WORK.fitt2

OUTFILE = ""N:\Downloads\SASFile\IndeterminatesExcluded\Fit statistics2.csv""

DBMS=CSV REPLACE;

RUN;

/* Export covariance parameter estimates table */

PROC EXPORT DATA= WORK.covparmestt2

OUTFILE = ""N:\Downloads\SASFile\IndeterminatesExcluded\Covariance parameter estimates2.csv""

DBMS=CSV REPLACE;

RUN;

/* MODEL 3 */

ods output ParameterEstimates=pet3 FitStatistics=fitt3 additionalestimates=addest3

CovMatParmEst=covparmestt3 ConvergenceStatus=convgstatt3 additionalestimates=addest3;

/* Run random effects logistic regression model for sensitivity and fixed model for specificity */

proc nlmixed data=dt cov tech=quanew lis=5 qpoints=10;

parms msens=2 mspec=1  s2usens=0 ;

logitp=(msens+usens)*sens+(mspec)*spec;

p = exp(logitp)/(1+exp(logitp));

model true ~ binomial(n,p);

random usens ~ normal([0],[s2usens]) subject=study_id out=randeffs;

estimate 'logLR+' log((exp(msens)/(1+exp(msens)))/(1-(exp(mspec)/(1+exp(mspec)))));

estimate 'logLR-' log((1-(exp(msens)/(1+exp(msens))))/(exp(mspec)/(1+exp(mspec))));

run;

/* Obtain summary sens and spec from the model 3*/

/* change the number if this is for a different model*/

data summary3;

set pet3;

if parameter = 'msens' then name = 'Sensitivity';

else if parameter = 'mspec' then name = 'Specificity';

if parameter = 'msens' or parameter ='mspec' then summary=100 * exp(estimate)/(1 + exp(estimate));

if parameter = 'msens' or parameter ='mspec' then summlower=100 * exp(lower)/(1 + exp(lower));

if parameter = 'msens' or parameter ='mspec' then summupper=100 *exp(upper)/(1 + exp(upper));

output;

run;

/* Obtain summary LR from the model 3 */

data summaryLR3;

set addest3;

summary=exp(estimate);

summlower=exp(lower);

summupper=exp(upper);

output;

run;

PROC EXPORT DATA= WORK.SUMMARY3

OUTFILE = ""N:\Downloads\SASFile\IndeterminatesExcluded\Summary3.csv""

DBMS=CSV REPLACE;

RUN;

/* Export parameter estimates table */

PROC EXPORT DATA= WORK.pet3

OUTFILE = ""N:\Downloads\SASFile\IndeterminatesExcluded\Parameter estimates3.csv""

DBMS=CSV REPLACE;

RUN;

/* Export the summary LR as an Excel .csv file */

PROC EXPORT DATA= WORK.SUMMARYLR3

OUTFILE = ""N:\Downloads\SASFile\IndeterminatesExcluded\SummaryLR3.csv""

DBMS=CSV REPLACE;

RUN;

/* Export Fit statistics table */

PROC EXPORT DATA= WORK.fitt3

OUTFILE = ""N:\Downloads\SASFile\IndeterminatesExcluded\Fit statistics3.csv""

DBMS=CSV REPLACE;

RUN;

/* Export covariance parameter estimates table */

PROC EXPORT DATA= WORK.covparmestt3

OUTFILE = ""N:\Downloads\SASFile\IndeterminatesExcluded\Covariance parameter estimates3.csv""

DBMS=CSV REPLACE;

RUN;

/* MODEL 4 */

ods output ParameterEstimates=pet4 FitStatistics=fitt4 additionalestimates=addest4

CovMatParmEst=covparmestt4 ConvergenceStatus=convgstatt4;

/* Run fixed effect logistic regression model for sensitivity and random effects model for specificity */

proc nlmixed data=dt cov tech=quanew lis=5 qpoints=10;

parms msens=2 mspec=1  s2uspec=0 ;

logitp=(msens)*sens+(mspec+uspec)*spec;

p = exp(logitp)/(1+exp(logitp));

model true ~ binomial(n,p);

random uspec ~ normal([0],[s2uspec]) subject=study_id out=randeffs;

estimate 'logLR+' log((exp(msens)/(1+exp(msens)))/(1-(exp(mspec)/(1+exp(mspec)))));

estimate 'logLR-' log((1-(exp(msens)/(1+exp(msens))))/(exp(mspec)/(1+exp(mspec))));

run;

/* Obtain summary sens and spec from the model 4*/

/* change the number if this is for a different model*/

data summary4;

set pet4;

if parameter = 'msens' then name = 'Sensitivity';

else if parameter = 'mspec' then name = 'Specificity';

if parameter = 'msens' or parameter ='mspec' then summary=100 * exp(estimate)/(1 + exp(estimate));

if parameter = 'msens' or parameter ='mspec' then summlower=100 * exp(lower)/(1 + exp(lower));

if parameter = 'msens' or parameter ='mspec' then summupper=100 *exp(upper)/(1 + exp(upper));

output;

run;

/* Obtain summary LR from the model 4 */

data summaryLR4;

set addest4;

summary=exp(estimate);

summlower=exp(lower);

summupper=exp(upper);

output;

run;

PROC EXPORT DATA= WORK.SUMMARY4

OUTFILE = ""N:\Downloads\SASFile\IndeterminatesExcluded\Summary4.csv""

DBMS=CSV REPLACE;

RUN;

/* Export parameter estimates table */

PROC EXPORT DATA= WORK.pet4

OUTFILE = ""N:\Downloads\SASFile\IndeterminatesExcluded\Parameter estimates4.csv""

DBMS=CSV REPLACE;

RUN;

/* Export the summary LR as an Excel .csv file */

PROC EXPORT DATA= WORK.SUMMARYLR4

OUTFILE = ""N:\Downloads\SASFile\IndeterminatesExcluded\SummaryLR4.csv""

DBMS=CSV REPLACE;

RUN;

/* Export Fit statistics table */

PROC EXPORT DATA= WORK.fitt4

OUTFILE = ""N:\Downloads\SASFile\IndeterminatesExcluded\Fit statistics4.csv""

DBMS=CSV REPLACE;

RUN;

/* Export covariance parameter estimates table */

PROC EXPORT DATA= WORK.covparmestt4

OUTFILE = ""N:\Downloads\SASFile\IndeterminatesExcluded\Covariance parameter estimates4.csv""

DBMS=CSV REPLACE;

RUN;

/* MODEL 5 */

ods output ParameterEstimates=pet5 FitStatistics=fitt5 additionalestimates=addest5

CovMatParmEst=covparmestt5 ConvergenceStatus=convgstatt5;

/* Run fixed effect logistic regression model for sensitivity and specificity */

proc nlmixed data=dt cov tech=quanew lis=5 qpoints=10;

parms msens=2 mspec=1;

logitp=(msens)*sens+(mspec)*spec;

p = exp(logitp)/(1+exp(logitp));

model true ~ binomial(n,p);

estimate 'logLR+' log((exp(msens)/(1+exp(msens)))/(1-(exp(mspec)/(1+exp(mspec)))));

estimate 'logLR-' log((1-(exp(msens)/(1+exp(msens))))/(exp(mspec)/(1+exp(mspec))));

run;

/* Obtain summary sens and spec from the model 5*/

/* change the number if this is for a different model*/

data summary5;

set pet5;

if parameter = 'msens' then name = 'Sensitivity';

else if parameter = 'mspec' then name = 'Specificity';

if parameter = 'msens' or parameter ='mspec' then summary=100 * exp(estimate)/(1 + exp(estimate));

if parameter = 'msens' or parameter ='mspec' then summlower=100 * exp(lower)/(1 + exp(lower));

if parameter = 'msens' or parameter ='mspec' then summupper=100 *exp(upper)/(1 + exp(upper));

output;

run;

/* Obtain summary LR from the model 5 */

data summaryLR5;

set addest5;

summary=exp(estimate);

summlower=exp(lower);

summupper=exp(upper);

output;

run;

PROC EXPORT DATA= WORK.SUMMARY5

OUTFILE = ""N:\Downloads\SASFile\IndeterminatesExcluded\Summary5.csv""

DBMS=CSV REPLACE;

RUN;

/* Export parameter estimates table */

PROC EXPORT DATA= WORK.pet5

OUTFILE = ""N:\Downloads\SASFile\IndeterminatesExcluded\Parameter estimates5.csv""

DBMS=CSV REPLACE;

RUN;

/* Export the summary LR as an Excel .csv file */

PROC EXPORT DATA= WORK.SUMMARYLR5

OUTFILE = ""N:\Downloads\SASFile\IndeterminatesExcluded\SummaryLR5.csv""

DBMS=CSV REPLACE;

RUN;

/* Export Fit statistics table */

PROC EXPORT DATA= WORK.fitt5

OUTFILE = ""N:\Downloads\SASFile\IndeterminatesExcluded\Fit statistics5.csv""

DBMS=CSV REPLACE;

RUN;

/* Export covariance parameter estimates table */

PROC EXPORT DATA= WORK.covparmestt5

OUTFILE = ""N:\Downloads\SASFile\IndeterminatesExcluded\Covariance parameter estimates5.csv""

DBMS=CSV REPLACE;

RUN;

/* MODEL 6 */

ods output ParameterEstimates=pet6 FitStatistics=fitt6 additionalestimates=addest6;

/* Run random effects logistic regression model for sensitivity only*/

proc nlmixed data=dt tech=quanew lis=5 qpoints=10;

parms msens=2 s2usens=0 ;

logitp=(msens+usens)*sens;

p = exp(logitp)/(1+exp(logitp));

model true ~ binomial(n,p);

random usens ~ normal([0],[s2usens]) subject=study_id out=randeffs;

/* logLR based on spec=1 */

estimate 'logLR-' log((1-(exp(msens)/(1+exp(msens))))) ;

run;

/* Obtain summary sens from the model 6 */

data summary6;

set pet6;

if parameter = 'msens' then name = 'Sensitivity';

if parameter = 'msens' then summary=100 * exp(estimate)/(1 + exp(estimate));

if parameter = 'msens' then summlower=100 * exp(lower)/(1 + exp(lower));

if parameter = 'msens' then summupper=100 *exp(upper)/(1 + exp(upper));

output;

run;

/* Obtain summary LR from the model 6 */

data summaryLR6;

set addest6;

summary=exp(estimate);

summlower=exp(lower);

summupper=exp(upper);

output;

run;

PROC EXPORT DATA= WORK.SUMMARY6

OUTFILE = ""N:\Downloads\SASFile\IndeterminatesExcluded\Summary6.csv""

DBMS=CSV REPLACE;

RUN;

/* Export parameter estimates table */

PROC EXPORT DATA= WORK.pet6

OUTFILE = ""N:\Downloads\SASFile\IndeterminatesExcluded\Parameter estimates6.csv""

DBMS=CSV REPLACE;

RUN;

/* Export the summary LR as an Excel .csv file */

PROC EXPORT DATA= WORK.SUMMARYLR6

OUTFILE = ""N:\Downloads\SASFile\IndeterminatesExcluded\SummaryLR6.csv""

DBMS=CSV REPLACE;

RUN;

/* Export Fit statistics table */

PROC EXPORT DATA= WORK.fitt6

OUTFILE = ""N:\Downloads\SASFile\IndeterminatesExcluded\Fit statistics6.csv""

DBMS=CSV REPLACE;

RUN;

"

**[^18^F]FDG PET**

data DiagnosticTestMetaAnalysis;

input Study_id TP FP FN TN;

datalines;

1 1 0 12 2

2 2 0 1 1

3 3 1 8 1

4 8 2 0 1

run;

/* Modify the dataset for the bivariate analysis */

data dt;

set DiagnosticTestMetaAnalysis;

sens=1; spec=0; true=tp; n=tp+fn; output;

sens=0; spec=1; true=tn; n=tn+fp; output;

run;

/* Ensure that both records for a study are clustered together */

proc sort data=dt;

by study_id ;

run;

/* MODEL 1 */

/* Save NLMIXED output in the following datasets*/

ods output ParameterEstimates=pet1 FitStatistics=fitt1 additionalestimates=addest1

CovMatParmEst=covparmestt1 ConvergenceStatus=convgstatt1;

/* Run the bivariate random effects logistic regression model for sensitivity and specificity */

/* The cov option requests that a covariance matrix is printed for all model parameter estimates.*/

proc nlmixed data=dt cov tech=quanew lis=5;

parms msens=2 mspec=1 s2usens=0 s2uspec=0 covsesp=0;

logitp=(msens+usens)*sens+(mspec+uspec)*spec;

p = exp(logitp)/(1+exp(logitp));

model true ~ binomial(n,p);

random usens uspec ~ normal([0,0],[s2usens,covsesp,s2uspec]) subject=study_id out=randeffs;

estimate 'logLR+' log((exp(msens)/(1+exp(msens)))/(1-(exp(mspec)/(1+exp(mspec)))));

estimate 'logLR-' log((1-(exp(msens)/(1+exp(msens))))/(exp(mspec)/(1+exp(mspec))));

run;

/* Obtain summary sens and spec from the model 1*/

/* change the number if this is for a different model*/

data summary1;

set pet1;

if parameter = 'msens' then name = 'Sensitivity';

else if parameter = 'mspec' then name = 'Specificity';

if parameter = 'msens' or parameter ='mspec' then summary=100 * exp(estimate)/(1 + exp(estimate));

if parameter = 'msens' or parameter ='mspec' then summlower=100 * exp(lower)/(1 + exp(lower));

if parameter = 'msens' or parameter ='mspec' then summupper=100 *exp(upper)/(1 + exp(upper));

output;

run;

/* Obtain summary LR from the model 1 */

data summaryLR1;

set addest1;

summary=exp(estimate);

summlower=exp(lower);

summupper=exp(upper);

output;

run;

PROC EXPORT DATA= WORK.SUMMARY1

OUTFILE = "N:\Downloads\Systematic review file\SASFile\IndeterminatesExcluded\Summary1.csv"

DBMS=CSV REPLACE;

RUN;

/* Export parameter estimates table */

PROC EXPORT DATA= WORK.pet1

OUTFILE = "N:\Downloads\Systematic review file\SASFile\IndeterminatesExcluded\Parameter estimates1.csv"

DBMS=CSV REPLACE;

RUN;

/* Export the summary LR as an Excel .csv file */

PROC EXPORT DATA= WORK.SUMMARYLR1

OUTFILE = "N:\Downloads\Systematic review file\SASFile\IndeterminatesExcluded\SummaryLR1.csv"

DBMS=CSV REPLACE;

RUN;

/* Export Fit statistics table */

PROC EXPORT DATA= WORK.fitt1

OUTFILE = "N:\Downloads\Systematic review file\SASFile\IndeterminatesExcluded\Fit statistics1.csv"

DBMS=CSV REPLACE;

RUN;

/* Export covariance parameter estimates table */

PROC EXPORT DATA= WORK.covparmestt1

OUTFILE = "N:\Downloads\Systematic review file\SASFile\IndeterminatesExcluded\Covariance parameter estimates1.csv"

DBMS=CSV REPLACE;

RUN;

/* MODEL 2 */

ods output ParameterEstimates=pet2 FitStatistics=fitt2 additionalestimates=addest2 CovMatParmEst=covparmestt2 ConvergenceStatus=convgstatt2;

/* Run univariate random effects logistic regression models for sensitivity and specificity, i.e., ignore the correlation */

proc nlmixed data=dt cov tech=quanew lis=5;

parms msens=2 mspec=1 s2usens=0 s2uspec=0 ;

logitp=(msens+usens)*sens+(mspec+uspec)*spec;

p = exp(logitp)/(1+exp(logitp));

model true ~ binomial(n,p);

random usens uspec ~ normal([0,0],[s2usens,0,s2uspec]) subject=study_id out=randeffs;

estimate 'logLR+' log((exp(msens)/(1+exp(msens)))/(1-(exp(mspec)/(1+exp(mspec)))));

estimate 'logLR-' log((1-(exp(msens)/(1+exp(msens))))/(exp(mspec)/(1+exp(mspec))));

run;

/* Obtain summary sens and spec from the model 2*/

/* change the number if this is for a different model*/

data summary2;

set pet2;

if parameter = 'msens' then name = 'Sensitivity';

else if parameter = 'mspec' then name = 'Specificity';

if parameter = 'msens' or parameter ='mspec' then summary=100 * exp(estimate)/(1 + exp(estimate));

if parameter = 'msens' or parameter ='mspec' then summlower=100 * exp(lower)/(1 + exp(lower));

if parameter = 'msens' or parameter ='mspec' then summupper=100 *exp(upper)/(1 + exp(upper));

output;

run;

/* Obtain summary LR from the model 2 */

data summaryLR2;

set addest2;

summary=exp(estimate);

summlower=exp(lower);

summupper=exp(upper);

output;

run;

PROC EXPORT DATA= WORK.SUMMARY2

OUTFILE = "N:\Downloads\Systematic review file\SASFile\IndeterminatesExcluded\Summary2.csv"

DBMS=CSV REPLACE;

RUN;

/* Export parameter estimates table */

PROC EXPORT DATA= WORK.pet2

OUTFILE = "N:\Downloads\Systematic review file\SASFile\IndeterminatesExcluded\Parameter estimates2.csv"

DBMS=CSV REPLACE;

RUN;

/* Export the summary LR as an Excel .csv file */

PROC EXPORT DATA= WORK.SUMMARYLR2

OUTFILE = "N:\Downloads\Systematic review file\SASFile\IndeterminatesExcluded\SummaryLR2.csv"

DBMS=CSV REPLACE;

RUN;

/* Export Fit statistics table */

PROC EXPORT DATA= WORK.fitt2

OUTFILE = "N:\Downloads\Systematic review file\SASFile\IndeterminatesExcluded\Fit statistics2.csv"

DBMS=CSV REPLACE;

RUN;

/* Export covariance parameter estimates table */

PROC EXPORT DATA= WORK.covparmestt2

OUTFILE = "N:\Downloads\Systematic review file\SASFile\IndeterminatesExcluded\Covariance parameter estimates2.csv"

DBMS=CSV REPLACE;

RUN;

/* MODEL 3 */

ods output ParameterEstimates=pet3 FitStatistics=fitt3 additionalestimates=addest3

CovMatParmEst=covparmestt3 ConvergenceStatus=convgstatt3 additionalestimates=addest3;

/* Run random effects logistic regression model for sensitivity and fixed model for specificity */

proc nlmixed data=dt cov tech=quanew lis=5 qpoints=10;

parms msens=2 mspec=1 s2usens=0 ;

logitp=(msens+usens)*sens+(mspec)*spec;

p = exp(logitp)/(1+exp(logitp));

model true ~ binomial(n,p);

random usens ~ normal([0],[s2usens]) subject=study_id out=randeffs;

estimate 'logLR+' log((exp(msens)/(1+exp(msens)))/(1-(exp(mspec)/(1+exp(mspec)))));

estimate 'logLR-' log((1-(exp(msens)/(1+exp(msens))))/(exp(mspec)/(1+exp(mspec))));

run;

/* Obtain summary sens and spec from the model 3*/

/* change the number if this is for a different model*/

data summary3;

set pet3;

if parameter = 'msens' then name = 'Sensitivity';

else if parameter = 'mspec' then name = 'Specificity';

if parameter = 'msens' or parameter ='mspec' then summary=100 * exp(estimate)/(1 + exp(estimate));

if parameter = 'msens' or parameter ='mspec' then summlower=100 * exp(lower)/(1 + exp(lower));

if parameter = 'msens' or parameter ='mspec' then summupper=100 *exp(upper)/(1 + exp(upper));

output;

run;

/* Obtain summary LR from the model 3 */

data summaryLR3;

set addest3;

summary=exp(estimate);

summlower=exp(lower);

summupper=exp(upper);

output;

run;

PROC EXPORT DATA= WORK.SUMMARY3

OUTFILE = "N:\Downloads\Systematic review file\SASFile\IndeterminatesExcluded\Summary3.csv"

DBMS=CSV REPLACE;

RUN;

/* Export parameter estimates table */

PROC EXPORT DATA= WORK.pet3

OUTFILE = "N:\Downloads\Systematic review file\SASFile\IndeterminatesExcluded\Parameter estimates3.csv"

DBMS=CSV REPLACE;

RUN;

/* Export the summary LR as an Excel .csv file */

PROC EXPORT DATA= WORK.SUMMARYLR3

OUTFILE = "N:\Downloads\Systematic review file\SASFile\IndeterminatesExcluded\SummaryLR3.csv"

DBMS=CSV REPLACE;

RUN;

/* Export Fit statistics table */

PROC EXPORT DATA= WORK.fitt3

OUTFILE = "N:\Downloads\Systematic review file\SASFile\IndeterminatesExcluded\Fit statistics3.csv"

DBMS=CSV REPLACE;

RUN;

/* Export covariance parameter estimates table */

PROC EXPORT DATA= WORK.covparmestt3

OUTFILE = "N:\Downloads\Systematic review file\SASFile\IndeterminatesExcluded\Covariance parameter estimates3.csv"

DBMS=CSV REPLACE;

RUN;

/* MODEL 4 */

ods output ParameterEstimates=pet4 FitStatistics=fitt4 additionalestimates=addest4

CovMatParmEst=covparmestt4 ConvergenceStatus=convgstatt4;

/* Run fixed effect logistic regression model for sensitivity and random effects model for specificity */

proc nlmixed data=dt cov tech=quanew lis=5 qpoints=10;

parms msens=2 mspec=1 s2uspec=0 ;

logitp=(msens)*sens+(mspec+uspec)*spec;

p = exp(logitp)/(1+exp(logitp));

model true ~ binomial(n,p);

random uspec ~ normal([0],[s2uspec]) subject=study_id out=randeffs;

estimate 'logLR+' log((exp(msens)/(1+exp(msens)))/(1-(exp(mspec)/(1+exp(mspec)))));

estimate 'logLR-' log((1-(exp(msens)/(1+exp(msens))))/(exp(mspec)/(1+exp(mspec))));

run;

/* Obtain summary sens and spec from the model 4*/

/* change the number if this is for a different model*/

data summary4;

set pet4;

if parameter = 'msens' then name = 'Sensitivity';

else if parameter = 'mspec' then name = 'Specificity';

if parameter = 'msens' or parameter ='mspec' then summary=100 * exp(estimate)/(1 + exp(estimate));

if parameter = 'msens' or parameter ='mspec' then summlower=100 * exp(lower)/(1 + exp(lower));

if parameter = 'msens' or parameter ='mspec' then summupper=100 *exp(upper)/(1 + exp(upper));

output;

run;

/* Obtain summary LR from the model 4 */

data summaryLR4;

set addest4;

summary=exp(estimate);

summlower=exp(lower);

summupper=exp(upper);

output;

run;

PROC EXPORT DATA= WORK.SUMMARY4

OUTFILE = "N:\Downloads\Systematic review file\SASFile\IndeterminatesExcluded\Summary4.csv"

DBMS=CSV REPLACE;

RUN;

/* Export parameter estimates table */

PROC EXPORT DATA= WORK.pet4

OUTFILE = "N:\Downloads\Systematic review file\SASFile\IndeterminatesExcluded\Parameter estimates4.csv"

DBMS=CSV REPLACE;

RUN;

/* Export the summary LR as an Excel .csv file */

PROC EXPORT DATA= WORK.SUMMARYLR4

OUTFILE = "N:\Downloads\Systematic review file\SASFile\IndeterminatesExcluded\SummaryLR4.csv"

DBMS=CSV REPLACE;

RUN;

/* Export Fit statistics table */

PROC EXPORT DATA= WORK.fitt4

OUTFILE = "N:\Downloads\Systematic review file\SASFile\IndeterminatesExcluded\Fit statistics4.csv"

DBMS=CSV REPLACE;

RUN;

/* Export covariance parameter estimates table */

PROC EXPORT DATA= WORK.covparmestt4

OUTFILE = "N:\Downloads\Systematic review file\SASFile\IndeterminatesExcluded\Covariance parameter estimates4.csv"

DBMS=CSV REPLACE;

RUN;

/* MODEL 5 */

ods output ParameterEstimates=pet5 FitStatistics=fitt5 additionalestimates=addest5

CovMatParmEst=covparmestt5 ConvergenceStatus=convgstatt5;

/* Run fixed effect logistic regression model for sensitivity and specificity */

proc nlmixed data=dt cov tech=quanew lis=5 qpoints=10;

parms msens=2 mspec=1;

logitp=(msens)*sens+(mspec)*spec;

p = exp(logitp)/(1+exp(logitp));

model true ~ binomial(n,p);

estimate 'logLR+' log((exp(msens)/(1+exp(msens)))/(1-(exp(mspec)/(1+exp(mspec)))));

estimate 'logLR-' log((1-(exp(msens)/(1+exp(msens))))/(exp(mspec)/(1+exp(mspec))));

run;

/* Obtain summary sens and spec from the model 5*/

/* change the number if this is for a different model*/

data summary5;

set pet5;

if parameter = 'msens' then name = 'Sensitivity';

else if parameter = 'mspec' then name = 'Specificity';

if parameter = 'msens' or parameter ='mspec' then summary=100 * exp(estimate)/(1 + exp(estimate));

if parameter = 'msens' or parameter ='mspec' then summlower=100 * exp(lower)/(1 + exp(lower));

if parameter = 'msens' or parameter ='mspec' then summupper=100 *exp(upper)/(1 + exp(upper));

output;

run;

/* Obtain summary LR from the model 5 */

data summaryLR5;

set addest5;

summary=exp(estimate);

summlower=exp(lower);

summupper=exp(upper);

output;

run;

PROC EXPORT DATA= WORK.SUMMARY5

OUTFILE = "N:\Downloads\Systematic review file\SASFile\IndeterminatesExcluded\Summary5.csv"

DBMS=CSV REPLACE;

RUN;

/* Export parameter estimates table */

PROC EXPORT DATA= WORK.pet5

OUTFILE = "N:\Downloads\Systematic review file\SASFile\IndeterminatesExcluded\Parameter estimates5.csv"

DBMS=CSV REPLACE;

RUN;

/* Export the summary LR as an Excel .csv file */

PROC EXPORT DATA= WORK.SUMMARYLR5

OUTFILE = "N:\Downloads\Systematic review file\SASFile\IndeterminatesExcluded\SummaryLR5.csv"

DBMS=CSV REPLACE;

RUN;

/* Export Fit statistics table */

PROC EXPORT DATA= WORK.fitt5

OUTFILE = "N:\Downloads\Systematic review file\SASFile\IndeterminatesExcluded\Fit statistics5.csv"

DBMS=CSV REPLACE;

RUN;

/* Export covariance parameter estimates table */

PROC EXPORT DATA= WORK.covparmestt5

OUTFILE = "N:\Downloads\Systematic review file\SASFile\IndeterminatesExcluded\Covariance parameter estimates5.csv"

DBMS=CSV REPLACE;

RUN;

/* MODEL 6 */

ods output ParameterEstimates=pet6 FitStatistics=fitt6 additionalestimates=addest6;

/* Run random effects logistic regression model for sensitivity only*/

proc nlmixed data=dt tech=quanew lis=5 qpoints=10;

parms msens=2 s2usens=0 ;

logitp=(msens+usens)*sens;

p = exp(logitp)/(1+exp(logitp));

model true ~ binomial(n,p);

random usens ~ normal([0],[s2usens]) subject=study_id out=randeffs;

/* logLR based on spec=1 */

estimate 'logLR-' log((1-(exp(msens)/(1+exp(msens))))) ;

run;

/* Obtain summary sens from the model 6 */

data summary6;

set pet6;

if parameter = 'msens' then name = 'Sensitivity';

if parameter = 'msens' then summary=100 * exp(estimate)/(1 + exp(estimate));

if parameter = 'msens' then summlower=100 * exp(lower)/(1 + exp(lower));

if parameter = 'msens' then summupper=100 *exp(upper)/(1 + exp(upper));

output;

run;

/* Obtain summary LR from the model 6 */

data summaryLR6;

set addest6;

summary=exp(estimate);

summlower=exp(lower);

summupper=exp(upper);

output;

run;

PROC EXPORT DATA= WORK.SUMMARY6

OUTFILE = "N:\Downloads\Systematic review file\SASFile\IndeterminatesExcluded\Summary6.csv"

DBMS=CSV REPLACE;

RUN;

/* Export parameter estimates table */

PROC EXPORT DATA= WORK.pet6

OUTFILE = "N:\Downloads\Systematic review file\SASFile\IndeterminatesExcluded\Parameter estimates6.csv"

DBMS=CSV REPLACE;

RUN;

/* Export the summary LR as an Excel .csv file */

PROC EXPORT DATA= WORK.SUMMARYLR6

OUTFILE = "N:\Downloads\Systematic review file\SASFile\IndeterminatesExcluded\SummaryLR6.csv"

DBMS=CSV REPLACE;

RUN;

/* Export Fit statistics table */

PROC EXPORT DATA= WORK.fitt6

OUTFILE = "N:\Downloads\Systematic review file\SASFile\IndeterminatesExcluded\Fit statistics6.csv"

DBMS=CSV REPLACE;

RUN;
